# Mismatch-Tuned Plasmonic Nanogap Networks on Block Copolymers for Ultrasensitive Nucleic Acid Quantification

**DOI:** 10.1021/acsnanoscienceau.5c00190

**Published:** 2026-04-14

**Authors:** Yizhou Zhang, Ying Du, Martin Spillmann, Kazuto Akagi, Jiukai Tang, Shivaprakash N. Ramakrishna, Guangyu Qiu, Jing Wang

**Affiliations:** † Advanced Institute for Materials Research (WPI-AIMR), 13101 Tohoku University, Sendai 980-8577, Japan; ‡ School of Environmental and Chemical Engineering, Shanghai University, Shanghai 200444, China; § Engineering Research Center of Optical Instrument and System, the Ministry of Education, Shanghai Key Laboratory of Modern Optical System, 12624 University of Shanghai for Science and Technology, Shanghai 200093, China; ∥ Institute of Environmental Engineering, 27219 ETH Zürich, Zürich 8093, Switzerland; ⊥ Laboratory for Building Energy Materials and Component, Empa, Swiss Federal Laboratories for Materials Science and Technology, Dübendorf 8600, Switzerland; # Department of Materials, 27219 ETH Zürich, Zürich 8093, Switzerland; ∇ Institute of Medical Robotics, School of Biomedical Engineering, 12474 Shanghai Jiao Tong University, Shanghai 200240, P. R. China; ○ School of Biomedical Engineering, Shanghai Jiao Tong University, Shanghai 200240, P. R. China

**Keywords:** LSPR, biosensing, block copolymer, hotspots, SARS-CoV-2

## Abstract

In localized surface plasmon resonance (LSPR), rational nanogap engineering in metallic nanostructures enables strong plasmonic coupling and efficient confinement of incident electromagnetic fields, thereby significantly enhancing optical responses for biosensing applications. Traditional approaches to fabricating small gaps have relied on localizing dielectric spacers between gold nanoparticles (AuNPs). However, doing so has encountered challenges in producing high-density, clean gaps across large surface areas. Here, we demonstrate a straightforward, self-assembly-guided method for the consistent fabrication of topologically anchored AuNPs featuring sub-5 nm nanogaps, arranged on a nanostructured block copolymer template. The solution-based method enables time-dependent tuning toward high plasmonic coupling density, resulting in an extensive number of hotspots, with an equivalent sensing enhancement factor (ESEF) exceeding that of thermally annealed gold nanoisland chips by 1 × 10^5^. Laser excitation of these densely packed AuNPs at their plasmonic resonance efficiently drives both nucleic acid hybridization and amplification-based cyclic fluorescence probe cleavage, enabling SARS-CoV-2 viral sequence quantification down to the attomolar level. Our results demonstrate that a carefully engineered template nanostructure and the AuNP diameter integrate plasmonic hotspots, target adsorption, thermoplasmonic heating, and signal transduction within a single platform. This facile strategy for densely packed hotspots offers a potentially scalable avenue for ultrasensitive biomolecular assays.

## Graphical abstract

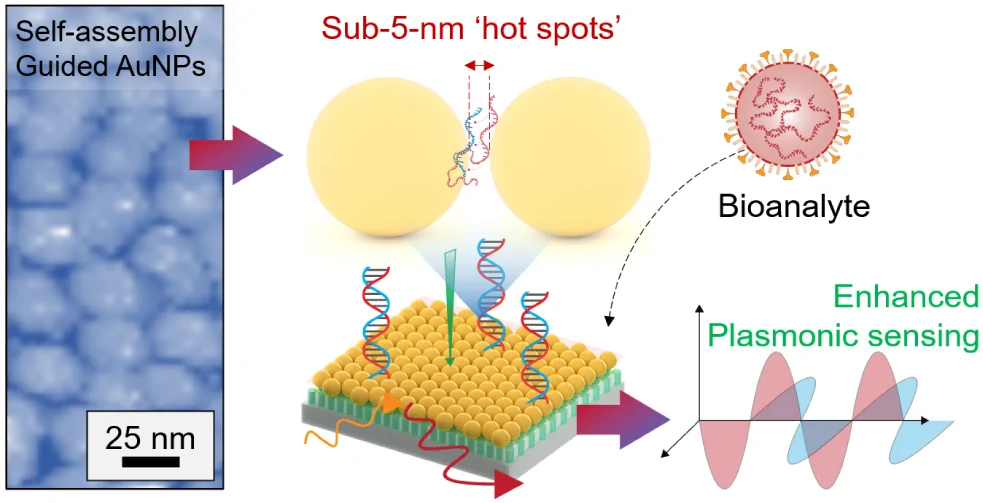

## Introduction

Quantitative biomolecular analysis is crucial for early disease diagnosis, public health surveillance, and environmental monitoring.^1^ Centralized laboratory diagnostics, such as reverse transcription polymerase chain reaction (RT-PCR), have demonstrated unparalleled accuracy in clinical and epidemiological applications.^2^ However, the requirement for skilled manpower, time-intensive processes, and well-equipped laboratories calls for decentralized biochemical analyses. Appropriately designed biosensors are poised to offer real-time, high-throughput analysis and pave the way for point-of-care (POC) with environmental sensing capabilities.^3^ Optical plasmonic devices based on nanomanufacturing have opened promising routes for detecting low-abundance analytes, offering the advantages of portability and cost-effectiveness.^4^

Localized surface plasmon resonance (LSPR), arising from the coherent oscillation of conduction electrons in metallic nanostructures, is highly sensitive to local changes in the refractive index (RI) and molecular binding events.^5^ Building upon these principles, recent work extends the LSPR sensing framework to universal molecular diagnostics and trace biomarker monitoring based on 40 nm gold nanoislands, which facilitated on-site quantitative risk analysis in viral-containing environments with femtomolar-level sensitivity.^6–8^ However, improved sensitivity is desired to enhance the efficacy of biological analytes at lower biomarker concentrations or from smaller volume samples. Sub-5 nm nanogaps at the interstitial junctions between metallic nanostructures can concentrate incident light, generating strongly enhanced plasmonic-coupled electromagnetic (EM) fields for light-matter interactions.^9–11^ In particular, the intensity of localized EM fields, or hotspots, possesses a nonuniform distribution across the surface and is highly sensitive to the size and geometry of the nanogaps between metallic bodies.^12–16^ The signal from analytes within these spatially confined volumes is significantly enhanced, making it particularly advantageous for highly sensitive plasmonic biosensing.^17^ However, this response enhancement is frequently limited by site-to-site variation arising from stoichiometric mixing of metallic nanostructures, leading to uncertainty and variability in the enhancement.^18,19^ While photolithography, electron-beam lithography (EBL), and focused ion beam (FIB) milling are effective for fabricating metal patterns, achieving precisely spaced gaps remains challenging for large-surface area fabrications.^20,21^

To achieve homogeneous LSPR enhancement across substrates, prior strategies rely on precise control over the uniform gap sizes achieved through the incorporation of dielectric spacers, including DNA,^22–25^ organic ligands,^26–28^ or polymers.^21,29–31^ This strategy is coupled with bottom-up processing to fabricate closely arranged nanoparticles. For example, Lu et al. created AuNPs superlattices, which involved interfacial assembly, subphase exchange, and free-floating ligand exchange to minimize defects and broken symmetry, thereby producing tunable gap sizes in the 1 nm range.^32^ However, the ligand-filled gaps hinder the effective accommodation of biomarkers, thereby compromising proper plasmonic sensing utility. Ideally, fabricating densely packed hotspots requires the guided anchoring of AuNPs with a uniform, small gap spacing, without excessive volumes of molecular spacers. Masson and coworkers demonstrated the formation of AuNP clusters on the surface brusher layer of the block copolymer with average gap distances exceeding 25 nm.^33,34^ The sensing capabilities of such systems have been exemplified by protein detection via LSPR,^35^ and by surface-enhanced Raman spectroscopy (SERS).^36–39^ To fabricate a high density of sub-5 nm gaps, however, deliberately selecting an appropriate guiding template that matches the size of the AuNPs is suitable.

Here, we introduce an LSPR substrate capable of real-time, attomolar-level detection for both amplification-based and amplification-free nucleic acid testing. The substrate uses intrinsically attached anchoring sites patterned from a self-assembled block copolymer thin film, allowing controlled and facile templating of AuNPs through a time-dependent solution immersion process. The topologically anchored AuNPs form sub-5 nm hotspot arrays through the carefully designed nanoparticle diameter and the nanostructured template spacing, which significantly enhances plasmonic activity under incident EM excitation. In particular, local EM fields enhancement factors exceeding 300 are achieved, and the finite-element method (FEM) calculation revealed that the EM field surface area integration factor, defined as the equivalent sensing enhancement factor (ESEF), is on the order of 10^5^-fold higher than that of previously reported gold nanoisland substrates.^6^ During the sensing, the self-assembled AuNP substrate was employed as a multifunctional medium that synergistically compiles densely packed hotspots, selective binding, heating, and signal transduction capabilities into a single platform. By leveraging laser-induced thermoplasmonics, the AuNPs array provides optimal local heating conditions for highly specific nucleic acid hybridization and catalyzes enzyme-based biomolecular reactions for signal amplification. The sensing system achieved limits of quantification (LoQ) ∼20 aM (12 copies μL^–1^) and 0.12 aM (0.072 copies μL^–1^) by direct viral nucleic acid oligomer detection and cyclic fluorescence probe cleavage detection, respectively, as well as direct binding detection of the WHO international standard for SARS-CoV-2 RNA 20/146 at a concentration of 0.88 copies μL^–1^. The mismatch-tuned plasmonic nanogap network presented here demonstrates a promising approach for achieving high analytical performance in LSPR-based sensing technologies.

## Results and Discussion

Connecting the precise self-assembly of the block copolymer with the homogeneous arrangement of localized EM fields between AuNPs enabled straightforward modulation of the LSPR sensing capability.^40,41^ We focused on a size-mismatch pairing mechanism using a hexagonal cylinder-forming poly­(styrene)-*b*-poly­(4-vinylpyridine), or PS_144_-*b*-P4VP_66.5_, with a polydispersity index of 1.04, as the structural template. This polymeric template has been extensively studied for its ability to anchor citric-acid–capped AuNPs through interactions with the P4VP domains.^34,36,42–44^ We initially spin-coated the block copolymer onto a piece of ∼4 cm^2^ BK glass, then immersed the nascent thin template in an ethanol bath for structural reorganization. The hexagonally structured thin nanofilm possessed a 25.2 nm center-to-center pore spacing with 10 nm diameter cylindrical P4VP domains oriented perpendicularly to the substrate. Monodispersed, citric acid-stabilized AuNPs were synthesized through a kinetically controlled seeded growth methodology targeting a controlled diameter of approximately 23 nm.^45^ The pore spacing was only a few nanometers larger than the AuNP diameter, providing a designed mismatch that promoted efficient spatial confinement. Therefore, sufficiently small gaps suitable for interparticle plasmon coupling were produced, as shown in Figure 1A. Because the density of plasmonic coupling was critical for enhanced local confinement, block copolymer thin films were immersed in an AuNP solution with unadjusted pH for a prolonged period, allowing for nanoparticle adsorption. In this manner, AuNPs were deposited in a densely packed array following the thermodynamically prescribed structural periodicities of block copolymers with a uniform spacing <5 nm through the prolonged 2.5 h solution immersion process.

**Figure 1:**
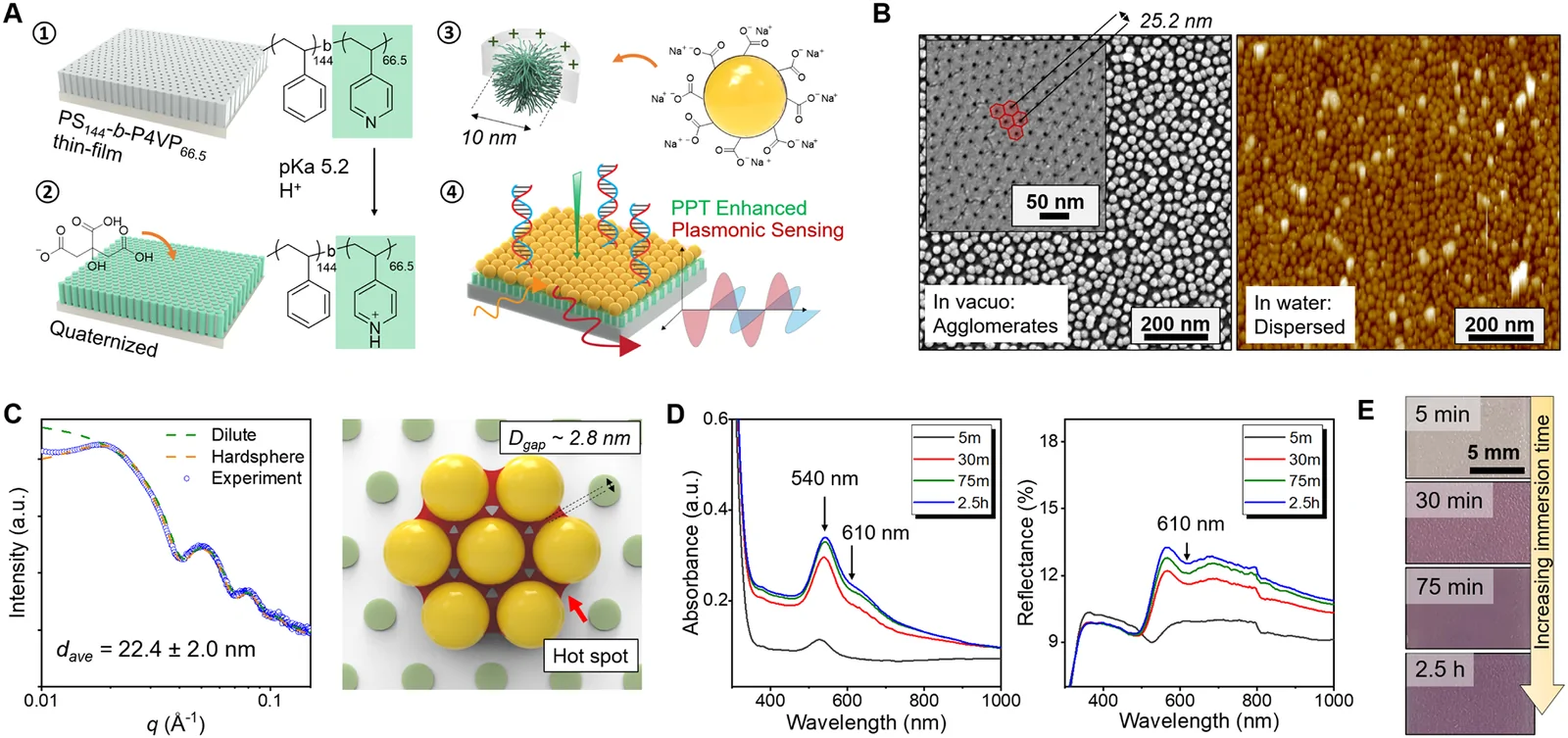
Surface-confined AuNP array on PS-*b*-P4VP nanofilms. (A) Schematics of the hexagonally nanostructured thin film with P4VP cylinders available for binding with citric acid-capped AuNPs. AuNPs were deposited on the PS-*b*-P4VP template by immersing the film in the solution for a predetermined amount of time. (B) The nanostructure of AuNPs assembly depended on the solvation environment. SEM analysis revealed there was aggregation of nanoparticles *in vacuo*, while AFM imaging demonstrates improved distribution of AuNPs in water. Inset: SEM micrograph of the block copolymer film showing a center-to-center pore spacing of 25.2 nm. (C) *In situ* SAXS pattern recorded from 22.4 nm diameter AuNPs attached on PS-*b*-P4VP film curved along a water-filled capillary. The estimated 2.8 nm interstitial junctions create locally enhanced EM fields. (D) UV–vis absorbance and reflectance spectra of AuNPs arrays prepared with different solution immersion times from 5 to 150 min, revealing that the templating registers a consistent plasmonic coupling between adjacent nanoparticles. (E) Photographs for the wetted chip prepared with increasing solution immersion time under white light illumination. The change in color manifests as an increase in the AuNPs density.

The extent of AuNP uptake and the anchoring morphology depended on the solution environment and nanoparticle size.^42^ The templating mechanism utilized confined hydrogen bonding and electrostatic interactions between citric acid residues and the polyelectrolyte brushes to achieve synergistically robust nanoparticle binding.^46,47^ In aqueous solution with neutral pH, P4VP was solvated and favored swelling to provide the anchoring surface area. The extent of nanoparticle adsorption was at the interplay between polymer brush swelling and AuNP to P4VP interactions.^42^ Assuming concentrated polymer brush packing, the hydrodynamic thickness (*h*) of surface-linked P4VP followed an N^0.8^ scaling behavior, estimated at ∼4.4 nm. The corresponding aerial density of P4VP was calculated as σ = ρhN_A_/*M*_n_, ∼0.43 chains per nm^2^, where ρ is the mass density of P4VP (1.14 g cm^–3^), *N*_A_ is Avogadro’s number, and *M*_n_ is the molecular weight of P4VP (6.99 kg mol^–1^).^48^ This assumption fell within the range of ∼0.1–0.5 chains per nm^2^ observed in previously studied grafted polymers, and a planar distribution of ∼20 nm spheres atop the brushes was expected.^42,49^ Additionally, the average chain-to-chain distance $D=\sqrt{1/\sigma }$ was only 1.5 nm, indicating the P4VP chains were tightly packed in a stretched chain conformation.^42^

Representative micrographs for densely packed AuNP surfaces from prolonged adsorption are shown in Figure 1B. Additional micrographs acquired from different immersion times are provided in Figure S1. Ideally, each 10 nm diameter P4VP column correspondingly accommodated one nanoparticle. The binding process yielded a homogeneous surface and was self-limiting as the binding area became less accessible due to steric hindrance, resulting in ∼8% of P4VP domains remaining unoccupied after 2.5 h of deposition (details in Figure S2). In the dried state, *i.e*., in vacuo, P4VP brushes were fully collapsed along the PS pore wall and allowed nanoparticles to aggregate, as observed in scanning electron microscopy (SEM) analysis. The emergence of aggregation was also a consequence of changing the dielectric constant (κ) surrounding citric acid-capped AuNPs from ∼78 in water to 1.0 in air. The change in the solvation environment resulted in the particles being distanced solely by the thickness of the citric acid residue. Though fully reversible, the apparent surface color changed from purple in water to plum slate in dry conditions manifested in increased aggregation (Figure S3). It should be noted that the interparticle gap while dry is thought to be inappropriately small for the nucleic acid sequence to fit for effective sensing. In contrast to the aggregated state, improved AuNP dispersion and their separation in DI water were further evidenced by tapping mode atomic force microscopy (AFM) performed in water. A detailed comparison is provided in Figure S4. In the aqueous environment, most AuNPs were positioned in-plane atop the block copolymer thin film without forming multilayer aggregations or being inserted into the polymeric matrix. By examining degree-1 homology (the “birth” and “death” events of ring structures) based on persistent homology, the arrangement of AuNPs was quantitatively analyzed. A comparison with the block copolymer template nanostructure revealed that the degree of ordering increased with longer immersion time or the saturation of the AuNP array (Figure S5). Therefore, the controlled adsorption of AuNPs in an adjusted solution environment ensured the reliability and precision of subsequent LSPR sensing experiments.

Subsequent structural characterization revealed a sub-5 nm gap between anchored AuNPs. Specifically, small-angle X-ray scattering (SAXS) was used to determine the anchored nanoparticle size using a lumen-coated capillary tube filled with water, with a schematic for the experiment shown in Figure S6. The fitting of the 1D curve from Figure 1C using the Igor Pro software package determined an AuNP diameter of 22.4 nm, with a dispersity of less than 10%. Therefore, by subtracting the averaged diameter of nanoparticles from the center-to-center pore spacing of 25.2 nm, an average interparticle distance of approximately 2.8 nm was obtained, with statistics shown in Figure S7. Similarly, the statistical distribution of gap sizes in the aqueous environment was determined from AFM micrographs to be **∼**2.9 nm. The nanostructured polymer template predominantly governed the binding behavior, ensuring a consistent AuNP neighboring distance independent of deposition time from 5 to 150 min. The consistency in coupling was further supported by absorbance and reflectance spectra of the anchored AuNP surface as a function of deposition time, as illustrated in Figure 1D. The transmittance spectra are shown in Figure S8. In particular, the adsorption spectra featured two peaks. The peak at higher photon energy corresponded to the transverse, coherent electron oscillation around the nanoparticle surface at ∼ 540 nm. The second peak, red-shifted to ∼610 nm, was attributed to the longitudinal coupling of surface plasmons and delocalization of the electric field within narrow crevices.^50,51^ Unlike other seeded growth methodologies where the coupling strength was kinetically determined, the reflectance demonstrates an unchanged wavelength of ∼610 nm and a full width at half-maximum (fwhm) of ∼60 nm. The consistent location of the absorbance and reflectance peaks unambiguously indicates a consistent plasmonic capacitive coupling distance. We surmise that the observed growth is attributed to an increased density of nanoparticles occupying the binding site rather than changes in coupling distance. For this reason, the photographs for a series of anchored AuNP surfaces with increasing growth times in Figure 1E, indicate an increase in nanoparticle density and reflectance cross-section with respect to time. It should be noted that the observed spectra are similar to prior reports on superlattices possessing gaps between 2 and 3 nm.^32,52^ However, the gap generated by the templating approach possesses a high density of free volumes available for the incoming analyte to occupy, as it contained predominantly water, rather than large-sized ligand molecules in between.

We further examined the plasmonic sensing behavior of the templated AuNP chip using a common-path interferometric LSPR system, as shown in Figure 2A. Namely, the sensing transduction was integrated with a normal incident laser illuminating the chip surface to excite closely packed nanoparticle antennae with sub-5 nm interparticle gaps, as shown in the magnified environmental AFM micrograph. The sensing beam was powered by a 33 μW broadband white LED source, positioned at a nominal incident angle of 72°, enabling the attenuated total reflection (ATR) configuration. In this configuration, the AuNPs interacted with the evanescent field rather than with propagating photons in the free space of the transmission mode. Signal to bulk RI variations in the absence of the laser provided preliminary information on the intrinsic sensitivity for the anchored AuNP chip surface. The phase response here was analyzed using the Windowed Fourier Transform (WFT) by directly measuring the real-time differential phase between the orthogonal polarizations, which is known for improved sensitivity of plasmonic sensors.^53^ The inclined angle of incidence and the prism coupling assigned a representative plasmonic resonance wavelength of ∼592 nm, and the phase changes were confined within a narrow wavelength region between 590 to 593 nm, as shown in Figure 2B.

**Figure 2:**
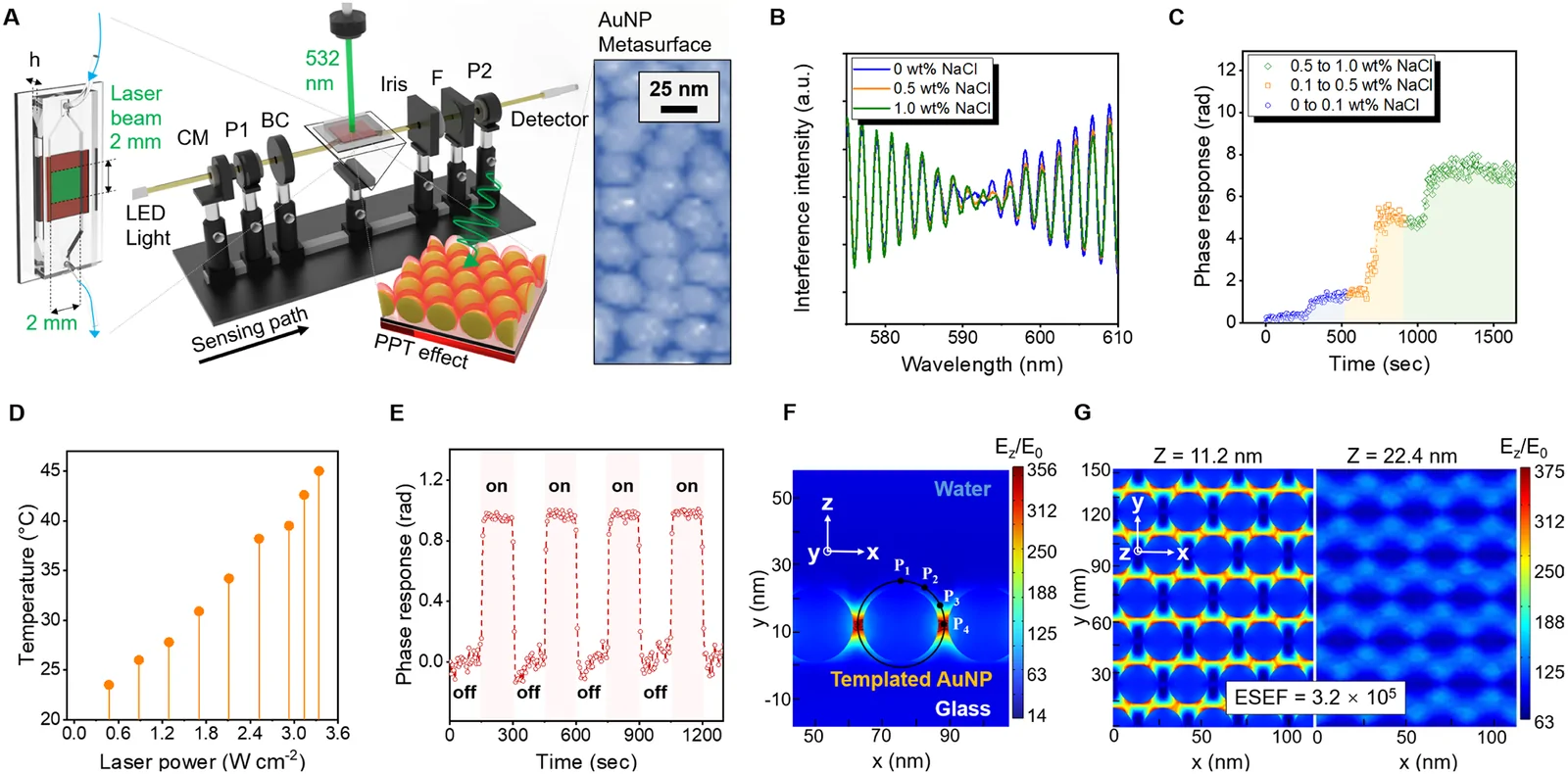
Integration of templated AuNP chip in a dual-functional plasmonic photothermal (PPT)-enhanced LSPR biosensing system. (A) Schematic illustration of the common-path LSPR phase sensing process, where a 532 nm laser diode uniformly illuminated the surface under normal incidence. The microfluidic channel height was ∼300 μm. Inset: magnified false-colored AFM image of the AuNP chip in water. (B) The interferogram spectra present a plasmonic resonance wavelength of ∼592 nm observed under the attenuated total reflection configuration. (C) Real-time phase shift recording in response to increasing NaCl solution concentration without affecting the AuNP anchoring position was analyzed using the broadband LED source. (D) Thermoplasmonic heating temperature response analysis correlated with the PPT effect as a function of laser power settings. (E) *In-situ* phase response demonstrates the rapid heating kinetics under periodic laser excitation. (F) An example of FEM calculated EM field distribution normalized to the incident laser illumination that consists of 22.4 nm diameter hexagonally arranged AuNPs in ∼2.8 nm spacing. Points P1 to P4 are located 1.4 nm from the AuNP surface. (G) Top view EM field distribution at different cross sections of the AuNP array.

For continuous microfluidic bioassays, the AuNP chip was fitted with a polydimethylsiloxane (PDMS) microfluidic window for the prospective solvent exchanges. The chip surface was first rinsed with excess DI water (RI = 1.3330) for >5 min to establish a stable baseline. Solutions of 0.1, 0.5, and 1.0 wt % NaCl were injected into the flow chamber to evaluate the bulk refractive index sensitivity (RIS), with the resulting spectra interferograms listed in Figure 2B. From 0 to 0.5 wt % (RI = 1.3339) of the NaCl solution, the temporal phase shift of 5.1 rads calculated in real-time is listed in Figure 2C. Additional data is provided in Figure S9. This corresponded to a RIS of 5600 ± 200 rad RIU^–1^, along with refractive index resolution (RIR) of 9.8 × 10^–6^ RIU at ∼595 nm, accounting for the standard deviation in blank measurement. Further increase in salt concentration to 1 wt % (RI = 1.3348) induced a phase shift to 7.5 rads. For reference, the chip prepared with a shorter nanoparticle solution immersion time of about 30 min exhibited lower surface AuNP coverage and larger average interparticle gaps, which reduced the response to NaCl solution, as shown in Figure S10. It should be noted that the P4VP moiety is stimuli-responsive and could undergo aggregation with respect to changes in the solution environment.^54–57^ The combined electrostatic interactions and hydrogen bonding within the polymer network minimized surface rearrangement during prolonged flow-through operations. A simple estimation indicated that in a solution containing NaCl, the P4VP moiety was not subject to transition to osmotic or salt-dominated regimes at concentrations below ∼260 mM (see calculation in Supporting Information).

Laser irradiation (λ = 532 nm) on surface-anchored AuNP chips served a dual role in facilitating plasmonic sensing. First, it enabled the transfer of photoexcited plasmons on a 4 mm^2^ homogenized square from the far field to the near field, thereby enhancing the LSPR sensitivity to NaCl solution, as exemplified by the difference between phase response on 0.1 wt % NaCl (Figure S10). A portion of this energy was dissipated as localized thermal energy through electron–phonon collisions or plasmonic photothermal (PPT) heating.^58^ The 532 nm excitation wavelength is closely aligned with the absorption peak of the AuNP chip. Therefore, with the elevated optical power at 2.7 W cm^–2^, PPT efficiently generated the required temperature necessary for nucleic acid hybridization and cyclic fluorescent-based amplification, which were utilized in the latter portion of this study. The *in situ* temperature calibration, or the photothermal temperature as a function of laser power, recorded up to 45 °C is depicted in Figure 2D.^58^ By modulating the laser power centered on the sensing transduction area, a stable temperature gradient responsible for RI variation was created. The corresponding real-time differential phase response, shown in Figure 2E, indicated the heating was instantaneous and reached the equilibrium within ∼10 s. The response was then retrieved to quantitatively correlate with the gradually increasing laser power, and the phase response to surface temperature correlation is further elaborated in Figure S11. Importantly, the excitation laser interacted with the LSPR antenna within the AuNP array for targets confined at the sub-5 nm features, generating a highly localized EM field that enhanced signal amplification and enabled highly sensitive biosensing applications.

In the realm of LSPR biosensing, the emphasis of response is placed on surface sensitivity instead of bulk sensitivity. This refers to the detection of the local RI in areas immediately adjacent to the analyte binding site, as the response from the surface binding is dominated by changes in the local EM field.^59,60^ The distribution of the electric field, therefore, plays a critical role in determining LSPR sensitivity.^61,62^ For example, Dorpe and coworkers proposed that the surface sensitivity is proportional to the product of bulk RI and the inverse of the decay length *l*_*d*_, meanwhile exhibiting an exponential decay with respect to *l*_*d*_.^61^ Following this calculation, the surface sensitivity reaches its maximum when the analyte and the receptor occupy the entire hotspot area. Such an optimal condition can be achieved when the metallic array possesses the exact spacing to fit in analytes, thus generating a highly confined surface EM field at the minimum spacing. For a detailed understanding of AuNP chip design parameters, we performed the simulation of the LSPR near fields using the FEM analysis by the COMSOL software package. In the model, the 532 nm laser radiation source generated a plane wave pulse and was irradiated perpendicularly onto the LSPR interface with an energy density of 2.7 W cm^–2^. The three-dimensional simulation of the electric field distribution at the interface is presented in Figure 2F and G. Areas of the maximum electric field intensity, or hotspots, were positioned within the 2.7 nm gaps between the adjacent particles. The maximum in the local EM field enhancement *|E/E*_0_ *|* was ∼360. This EM field enhancement is considerably higher than that of the self-assembled AuNP array prepared from block copolymer micelle lithography and the gold mushroom arrays.^21,63^ For comparison, the electric field distribution of 40 nm AuNPs 10 nm apart in a quadrilateral configuration with a similar layout to the thermally annealed surface is displayed in Figure S12, which shows an electric field enhancement factor *E/E*_0_ of ∼3.

Based on the near-field simulation results, the integration of electric field intensity over the surface area provided a quantitative measure of the equivalent sensing enhancement factor (ESEF), considering both the surface area and the EM amplitude of hotspots, as detailed in the Supporting Information. The ESEF value for the templated configuration depicted in Figure 2F is 3.2 × 10^5^. This value contrasts with the ESEF of ∼5 calculated under the assumption of thermally annealed geometry, shown in Figure S13. The significant difference in ESEF values arose from the impact of close-packed AuNP arrangement on the local electric field enhancement, in which a sub-5 nm gap distance and higher density of smaller diameter AuNPs contributed to the substantially confined EM field across the biomolecule binding interface. Figure S13 also presents a schematic illustration showing the hybridization of nucleic acid within the confined volume, and highlights the critical roles of nanoparticle diameters and spacings on ESEF. Specifically, hexagonally arranged AuNPs with a diameter of ∼22 nm produced more hotspots that exhibited a significantly stronger plasmonic near-field than the quadrilaterally arranged particle with diameters of 40 nm. Meanwhile, a smaller interparticle gap enhanced the plasmonic coupling effect and generated a stronger ESEF. Given that the effective persistent length of long single-stranded DNA (ssDNA) and single-stranded RNA (ssRNA) is ∼2.0 nm,^64,65^ the 2.8 nm interparticle gaps were well suited to accommodate the full sequence. We surmise that the appropriately spaced gap provided sufficient free volumes for the hybridized target-receptor sequence to accommodate. ESEF mapping further indicates that signal enhancement depends on both the number of plasmonic hotspots and the interparticle gap distance. With the present PS-*b*-P4VP template, however, further reduction of the gap spacing by modulating AuNP size, such as in a perfectly aligned, close-packed configuration with gaps below 1 nm, is not expected to significantly further improve the ESEF, as shown in Figure S13F. Instead, such extreme confinement may render target binding events inaccessible and introduce challenges in achieving homogeneous AuNP deposition across the array.^42^

Building upon the plasmonic near-field simulation, LSPR biosensing capability was experimentally evaluated by hybridizing the SARS-CoV-2 genome sequence with surface functionalized oligonucleotide receptors, as shown in Figure 3A. In order to develop a highly specific sequence for targeted hybridization, 10 μmol of freshly prepared synthetic receptor (Receptor-1) comprising 21 base pairs complementary to the ORF-1ab of the SARS-CoV-2 genome, terminated with the thiolated 5′ end, was continuously injected into the microfluidic biosensing channel. The genomic information is provided in Figure S14. The functionalization process was maintained for 60 min in the absence of PPT heating, allowing the citrate-to-thiol exchange to covalently functionalize the AuNPs at room temperature. Real-time monitoring of exemplary surface ligand exchange and functionalization is shown in Figure S15, indicating the reaction was kinetically limited compared with the bare gold surface, as the thiols were coattached on the surface with preadsorbed citrate on a mixed layer of AuNPs.^6^ Since additional intermolecular interactions such as steric-related and chelating effects were accounted for during the monolayer formation, the maximum surface density of the attached receptor was considerably lower than the self-assembled monolayer on bare gold, contributing to a lower saturation concentration of binding shown in the later paragraph.^66,67^

**Figure 3:**
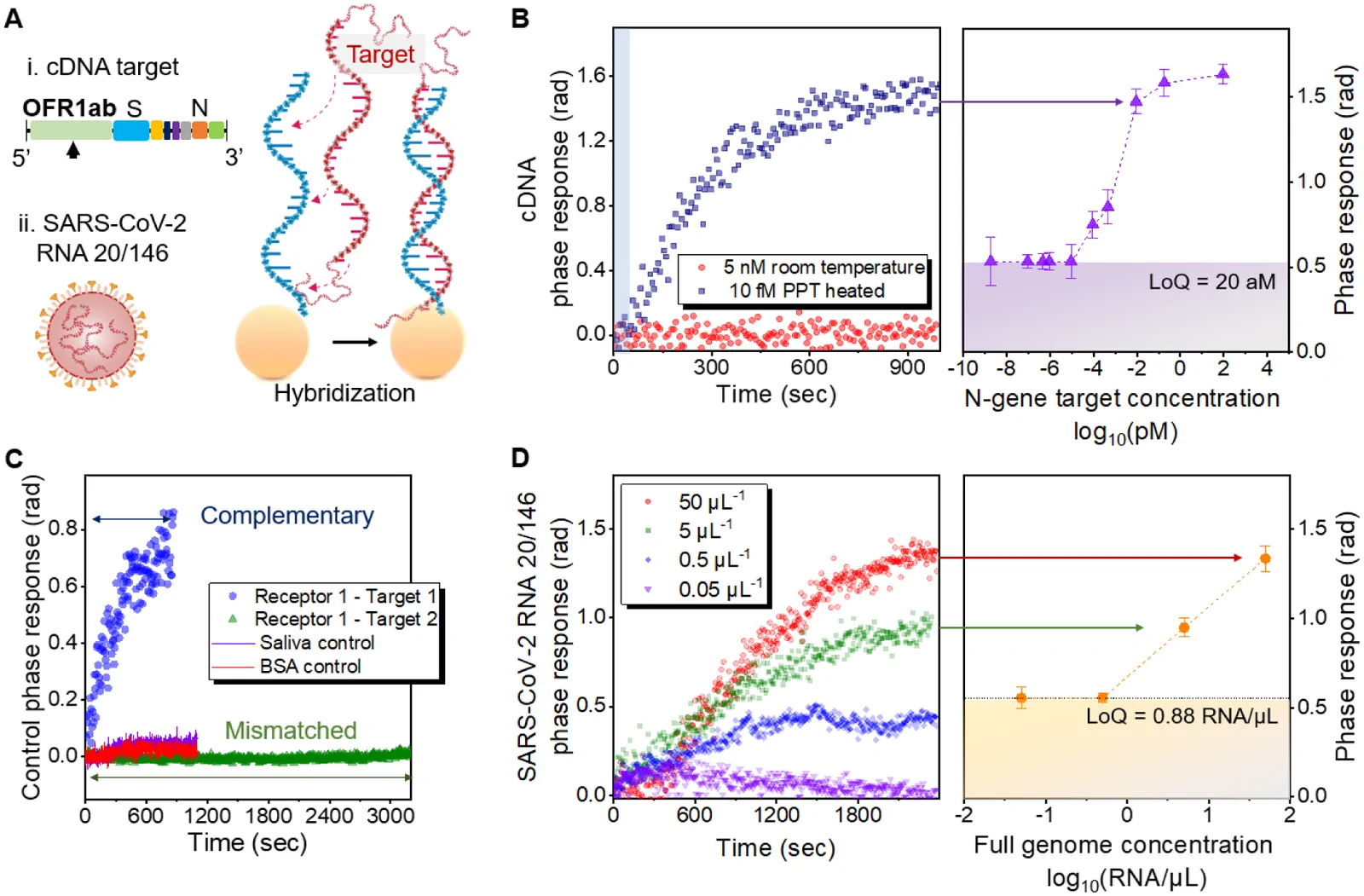
LSPR response analysis toward model nucleic acid sequences. (A) ORF-1ab, and the WHO international standard for SARS-CoV-2 RNA 20/146 were selected for photothermal hybridization with their complementary strands on surface functionalized AuNPs. (B) Comparative real-time hybridization of the ORF-1ab sequence with and without PPT enhancements. The response was pointed toward the complete thermoplasmonic bioassay, featuring saturation at ∼1 pM. In many instances, responses were below the calculated LoQ of the plasmonic system, which were adjusted up to 20 aM. (C) Comparison of PPT-enhanced hybridization between a fully complementary strand Target-1 and a mismatched sequence Target-2, with 0.1 wt % BSA and 100×-diluted saliva included as reference controls. (D) The real-time response curve of SARS-COV-2 RNA 20/146 across a range of concentrations. Responses below the calculated LoQ were adjusted up to 0.88 copies per μL.

The biosensing was preliminarily conducted using fully matching strands to examine the hybridization process with and without the PPT heating effect (Figure 3A). Here, the implication of temperature on sensing was examined using a 10 fM Target-1 comprised of 67 base pair sequences from the ORF-1ab, and detailed hybridization behavior was monitored through phase response. The sensing chamber was flushed with excess nuclease-free water with the thermoplasmonic laser activated at a laser power of 2.7 W cm^–2^, achieving a steady state baseline under a PPT heated temperature of 41 °C. Thereafter, the recording of the LSPR response began within 30 s after the introduction of the target, in which the plateaued response of 1.5 rads was documented after 900 s into the hybridization, as displayed in Figure 3B. In contrast, hybridization without the PPT heating at a 5 nM target concentration exhibited a negligible response. The reduced response, albeit in a higher target concentration, suggested a suppression of the binding event, likely related to the electrostatic interactions between the phosphate groups of the nucleic acid and the citric acid residue on the AuNP. This control experiment demonstrated that thermoplasmonic activation by PPT plays a critical role in enabling the ultrasensitive nucleic acid detection.

Detailed PPT-enhanced hybridization bioassays involving PPT heating are shown in Figure 3B, where binding was induced by flushing ORF-1ab Target-1 sequence across the functionalized AuNP array at a flow rate of 16 μL min^–1^ for 15 min, over a range of concentrations from 2 zM to 100 pM. The full range of responses is listed in Figure S16. Specifically, saturation of the LSPR response was observed at concentrations above 0.2 pM, equivalent to 12 pmol m^–2^ on the chip surface area. The limit of quantification (LoQ) was estimated from the sum of the nuclease-free water measurements with ten times the standard deviation, as demonstrated by the shaded purple area in the plot. The LoQ indicated that the templated AuNP chip offered a dynamic detection range from 20 aM to 0.2 pM. In practical terms, this LoQ can be translated to ∼2900 copies of the ORF-1ab segment in a 240 μL analyte sample. Therefore, the sensitivity generated by narrow spacing and strong EM field confinement surpassed that of thermal annealed AuNP chips, yielding a target concentration at the midpoint phase response that is ∼ 1 × 10^5^ lower, consistent with the corresponding difference in ESEF factors (∼1 × 10^5^).^6^ Hence, the difference in the calculated ESEF is consistent with the observed improvement in experimental detection sensitivity, supporting the structure–performance relationship established by the mismatch-tuned AuNP array. More importantly, the enhanced sensitivity was further complemented by the PPT heating, which effectively suppresses false-positives, as demonstrated by the experiment using mismatched sequences. At elevated temperatures, the system effectively discriminated against the mismatched ORF-1ab sequence, Target-2, in which 18 of 22 bases in Receptor-1 were mismatched. No hybridization was observed at a concentration of 10 fM even after an incubation period of 1 h (Figure 3C). This behavior is consistent with our previous observations that hybridization between the receptor and the RNA-dependent RNA polymerase (RdRp)-SARS sequence is fully suppressed to the blank baseline, in contrast to the strong binding observed with RdRp-COVID,^6–8^ whereas the combination of PPT enhancement with AuNPs confined coupling can serve as a highly sensitive and selective method for detecting trace analyte concentrations. Meanwhile, the negligible responses observed for the representative protein interferent, 0.1 wt % bovine serum albumin (BSA) solution, and the 100×-diluted saliva control indicate minimal nonspecific adsorption and good tolerance to complex biological matrices under assay-relevant conditions, with potential effects of ionic strength, mucin-rich backgrounds, proteins, and enzymatic degradation products on overall surface interactions. The combination of PPT enhancement with AuNPs confined coupling can serve as a highly sensitive method for detecting trace analyte concentrations while minimizing the rate of false positives and nonspecific interference.

Understanding the direct binding behavior between the close-packed AuNP chip and the full SARS-CoV-2 genome enabled direct comparison of the sensing efficiency with other nucleic acid testing protocols. In comparison with the ORF-1ab, the full SARS-CoV-2 genome was expected to be detectable at even lower concentrations and to produce a larger signal, as it contains 29.9 × 10^3^ base pairs, which is nearly 450 times the length of the Target-1. To elucidate the potential of label-free sensing, we assessed the response using the first WHO International Standard RNA in Nucleic Acid Amplification Technique (NAAT)-based assays, a target consistent with the acid-heat-inactivated England/02/2020 isolate of SARS-CoV-2. Figure 3D illustrates the in situ phase response to RNA at concentrations ranging from 0.05 to 50 copies per μL, which were introduced across the functionalized AuNP array for ∼40 min at a flow rate of 16 μL min^–1^. The LoQ was determined to be 0.88 copies per μL. Since the full sequence is substantially larger than oligomers, the probability of the target and receptor aligning correctly for binding within the 2.8 nm confinement is considerably reduced due to increased spatial confinement, despite our previous study has demonstrated the potential of enhanced target accumulation toward plasmonic hotspots in such optofluidic sensing systems.^68–70^ Nevertheless, the experimentally determined sensing limit based on real-time responses using the full genome is on par with that of literature documented RT-qPCR, which typically displays a limit of detection (LoD) of ∼0.1 copies per μL, as well as with our previously measured Target 1 (N1) sequences using SYBR Green–based RT-qPCR, and loop-mediated isothermal amplification (LAMP) of ∼2 copies per μL.^8,71,72^ The achieved sensitivity is also comparable to the most sensitive rapid antigen tests.^72^ The full-scale LSPR response for the direct full SARS-CoV-2 genome nucleic acid binding experiment is displayed in Figure S16.

While direct hybridization provided an efficient sensing approach through the templated AuNP chip, it falls short of fully exploiting the enhanced coupling potential within the sub-5 nm gaps. We further explored its sensing application to include a self-validating biosensing readout with cyclic-gain-assisted amplification for further enhanced nucleic acid sensitivity with minimized nonspecific interference.^7^ This was achieved by coupling biological reactions with plasmonic detection through a dual-mode transducing pathway, which was promoted by biological enzymatic activities, as shown in Figure 4A. By utilizing the heating and EM field-enhancing capabilities of the templated AuNP chip, we integrated the functionalities of signal transduction and fluorescent enhancement into a single sensing platform. An alternate sequence of the synthetic ORF-1ab of the SARS-CoV-2 genome with a length of 45 nucleic acids, Target-2, a mismatch of Receptor-1, was selected. The dual sensing operation began with the PPT-induced hybridization after immobilizing thiolated Receptor-2 on the AuNP surface. The response from direct binding mode across similar target concentrations and the same flow conditions is shown in Figure S17. The similar saturation behavior observed for Target-1 and Target-2 at ∼1 pM indicates that the surface coverage of the immobilized thiolated receptor is reproducible.

**Figure 4:**
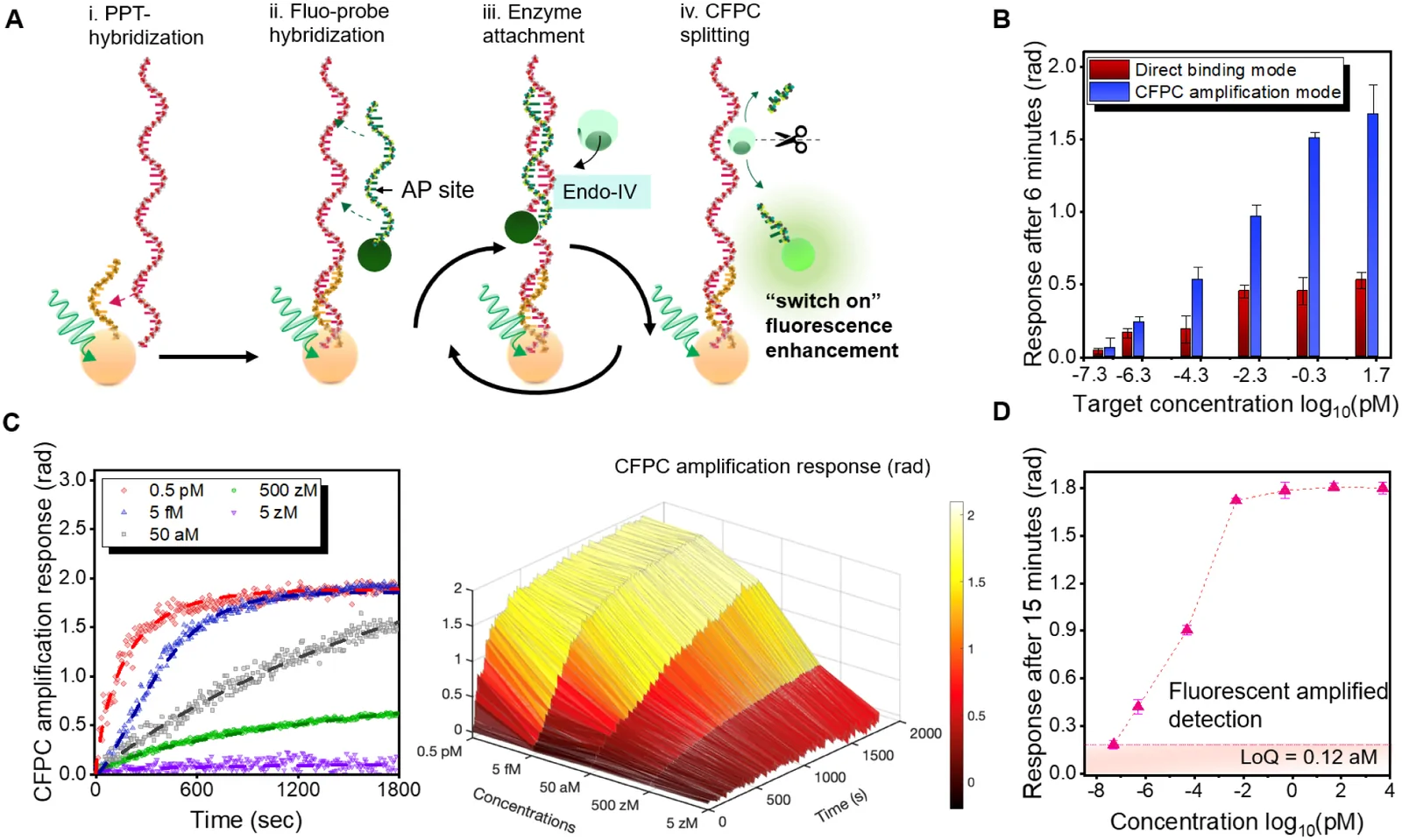
Amplification sensing via dual-mode fluorescence transduction pathway. (A) Schematic illustrating LSPR sensing augmented by PPT-assisted CFPC. Initial quantification utilized direct hybridization, which was followed by the hybridization of the AP-site-modified fluorescent probe and the introduction of the Endo-IV enzyme. Subsequent cleavage of the fluorophore from the quencher activated the fluorescence signal, thereby amplifying and accumulating LSPR responses. (B) Direct comparison of response kinetics between the CFPC amplification mode and direct binding mode within the first 6 min detection window. (C) Real-time amplification-based bioassay and mapping of the CFPC amplification mode readout, where the response was accumulated through resonant energy transduction between the binding-cleavage-dissociation fluorescence emission and plasmonic detection. (D) The calibration curve of the target sequence concentrations using the CFPC pathway. Responses below the calculated LoQ were adjusted up to 0.12 aM.

In the presence of Endonuclease IV, the fluorescent oligo-probe tagged with an ATTO532 fluorophore at its 5′ end enabled amplification through cyclic fluorescence probe cleavage (CFPC). Endonuclease IV is a site-specific nuclease that bisects the probe at the synthetic apurinic/apyrimidinic (AP) site into a 5′-fluorophore and a 3′-quencher terminus, with melting temperatures at 34 and 30 °C, respectively. Moreover, the PPT effect was confined to the two-dimensional AuNP array, as unnecessary enzymatic activity in bulk was limited. In this manner, under consistent 532 nm laser excitation, the fluorescence probes underwent cyclic cleavage, and the released fluorophores were accumulated adjacent to the strongly coupled near field.^68^ This led to continuous emission of low-energy photons, which triggered and aggregated LSPR, acting as a “switch on” signal that further amplified the responses. Overall, this method offered two sets of sensitive but independent readouts within a reasonable detection cycle, *e.g*., less than 20 min in total in the current study. Compared with the direct binding mode, the CFPC amplification mode demonstrated superior sensitivity and generated a stronger response within 6 min after initiation of the sensing process, in which the readout from amplification outperformed hybridization and self-validated the initial response, as illustrated in Figure 4B. Owing to the repeated release of fluorescence from a single hybridization event, the CFPC amplification mode achieves a dynamic range spanning *c.a*. seven orders of magnitude, substantially exceeding the dynamic range of four orders of magnitude of the direct binding mode, which may be constrained by hybridization kinetics.

Full responses from cyclical binding-cleavage-dissociation and the detection mapping are shown in Figure 4C. The gain-assisted signal increased linearly but quickly reached saturation at fM-level concentrations once the fluorophores were completely released. In contrast, at lower target concentrations, the fluorophores supply remained abundant, allowing a sustained rise in response. The complete LSPR sensing readout for amplification is shown in Figure S18. Using a similar LoQ calculation noted in direct hybridization sensing, the CFPC amplification mode determined nucleic acid concentration down to 0.12 aM, shown in Figure 4D, representing a ∼980-fold improvement compared to the direct binding mode LoQ of 117 aM. This heightened sensitivity rivaled that of RT-LAMP and fluorescence-based methods and Specific High Sensitivity Enzymatic Reporter UnLOCKing (SHERLOCK), yet remained compact enough to be integrated into a point-of-care system with a small form factor.^73,74^ Detailed SARS-CoV-2 detection performance metrics are summarized in Supplementary Tables S1 and S2. Overall, the combination of close-packed AuNPs, hotspot enhancement, and signal amplification offered strong potential for advancing portable biosensing devices with simple optical components. This was enabled by the precise fabrication of sub-5 nm gaps using a self-assembled template, effectively coupled with enhanced ESEF design, providing a practical route for building various rapid and sensitive optical detection pathways.

## Conclusion

In conclusion, a hotspots-enriched plasmonic sensing chip was fabricated by guiding a dimensionally mismatched assembly of AuNPs on the nanostructured block copolymer template, achieving interparticle spacings of <5 nm. The topologically anchored nanoparticles were confined to a homogeneous array of densely confined localized plasmonic EM fields when irradiated by light, offering robust LSPR enhancement. The arrangement of AuNPs contributed a surface area integrated ESEF that was ∼1× 10^5^ times greater than the thermally annealed AuNPs we previously reported. Incidence at two different angles excited plasmonic resonances of PPT and LSPR, which transformed the templated AuNP chip into a multifunctional platform that facilitated interfacial heating, selective nucleic acid adsorption, signal transduction, and readout enhancement. These synergistic mechanisms enabled real-time hybridization and self-validating fluorescence amplification with the limit of quantification down to the atto-molar level. Furthermore, the proposed AuNP chip can be tailored further through the polymer template design, including the implementation of alternative block copolymer chemistries, as well as by tuning the AuNP solution environment and the diameter of the nanoparticles, to engineer localized plasmonic near-fields. Meanwhile, maintaining uniform nanoparticle loading and interparticle spacing over very large substrate areas may require further optimization. These aspects represent opportunities for future process improvement, such as adjusting AuNP concentration, temperature, or implementing flow-assisted deposition strategies. While not within the primary focus of this study, the solution processing technique employed here offers potential for adaptation to scalable LSPR substrate manufacturing. Given their facile fabrication and the scope of tunability, these templated AuNP chips could position LSPR as a preferred option for providing a reliable and easy-to-implement sensing platform for the efficient and rapid real-time biomarkers quantification, including not only nucleic acids but also peptides, antibodies, and extracellular vesicles. A comprehensive evaluation using clinical samples, such as patient nasopharyngeal swabs or saliva, will be required to support its diagnostic performance in real-world conditions and to bridge fundamental plasmonic research with practical diagnostic applications.

## Materials and Methods

### Materials

All chemicals for this study were purchased from Merck and used as received unless otherwise noted. The diblock copolymer poly­(styrene)-*b*-poly­(4-vinylpyridine) (PS_144_-*b*-P4VP_66.5_) with a number-average molecular weight of *M*_*n*_ = 22 kg mol^–1^, a polydispersity index of 1.04, and weight fractions of *w*_*PS*_ = 0.68 and *w*_*P4VP*_ = 0.32, respectively, was purchased from Polymer Source, Inc. and used without further purification. Oligonucleotides targeting the ORF1ab regions, including the oligonucleotide targets, their corresponding thiol-cDNA receptors, and the fluorescent probe containing a fluorophore-quencher pair, were custom-synthesized by Microsynth AG. Nuclease-free water (Thermo Fisher Scientific) was used to dilute synthetic cDNAs and the World Health Organization (WHO) SARS-CoV-2 standard in the sensing experiments.

### Fabrication of Templated AuNP Chips

The polymer casting solution was prepared by dissolving 0.5% (by weight) of PS_144_-*b*-P4VP_66.5_ in toluene until a homogeneous solution was observed, followed by degassing in a sonicator. Block copolymer films were spin-coated on ∼4 cm^2^ BK7 glasses using a WS-650-8B spin coater (Laurell), which supports substrate diameters up to 20 cm, although only 2 cm-scale substrates were used in this study. Spin coating was performed at 2000 rpm for 60 s in a clean room with regulated temperature and humidity. Subsequently, the polymer films were immersed in a bath of ethanol for 30 min, which initiated surface reconstruction and resulted in a self-assembled surface with an average pore-to-pore spacing of 25.2 nm for templating.

Uniform 22.4 nm gold nanoparticles (AuNPs) were synthesized using a kinetically controlled seeded growth methodology. Initially, gold seeds were prepared by transferring 1 mL of 25 mM HAuCl_4_ precursor solution into an aqueous bath of 150 mL, boiling 2.2 mM sodium citrate solution. Subsequently, AuNPs were allowed to undergo a 4-step growth reaction involving the sequential addition of 1 mL 60 mM sodium citrate and 1 mL of 25 mM HAuCl_4_ in the 90 °C seed solution with a time delay of 2 min. In some cases where larger nanoparticles were targeted, the growth procedure was extended to 7 successive generations of AuNPs using the same reaction protocol. Solution pH was monitored using the pH-indicator strips.

Densely packed AuNP arrays were prepared by allowing citrate-functionalized nanoparticles to attach to P4VP-lined walls through solution immersion. Specifically, block copolymer films were submerged in the nanoparticle solutions for a predetermined time, and then were rinsed with excess deionized (DI) water. Subsequently, the AuNPs-attached BK glasses were installed with a customized poly­(dimethylsiloxane) microfluidic channel measuring 5 × 2 mm, featuring a sample chamber with a height of 300 μm.

### Block Copolymer Thin Film and AuNP Characterization

A Magellan 400 (Thermo Fisher Scientific) Extreme High-Resolution Field Emission Gun–Scanning Electron Microscope (XHR-FEG-SEM) was used to characterize the surface morphology of the AuNPs sensor chips. Samples were mounted on a standard pin sub using conductive silver paste and sputter-coated with ∼1.5 nm of Iridium. Images were acquired at an accelerating voltage of 10 kV and a working distance of 4 mm thereafter. Micrographs were analyzed using the ImageJ software package, where the number of particles was quantified in a given surface area. AFM imaging was performed on a Dimension ICON system (Bruker, Santa Barbara, USA) in PeakForce Tapping mode under milli-Q water using a fluid cell. PEAKFORCE-HIRS–F-B (Bruker probes) cantilever was employed, with a set point force of approximately 0.4 nN during imaging.

Small-angle X-ray scattering (SAXS) was conducted using a Xeuss 3.0 (Xenocs) system equipped with a Genix 3D Cu source with a wavelength of λ = 1.54 Å, with scattering data collected under a range of accessible scattering vectors (*q*) from 1.0 × 10^–2^ to 3.1 Å^–1^. To prepare samples with a consistent solvent environment presented in the sensing setup, AuNPs were immobilized on the lumen side of a capillary tube dip-coated with block copolymer thin film. The capillary was filled with deionized water, and both ends were securely sealed with epoxy resin. The 2D scattering pattern was acquired with the line-eraser mode using silver behenate as the standard for calibration. Scattering patterns were then azimuthally integrated by employing the Foxtrot software package for analyzing the scattering intensity (*I*) as a function of *q* = 4π sin­(θ)/λ, where θ is half of the scattering angle. The SAXS data were subsequently fitted using the Igor Pro software package to determine the nanoparticle diameter.

Reflectance and transmittance spectra were obtained using a Shimadzu UV–vis 3600 spectrophotometer equipped with an integrating sphere. For all measurements, the templated AuNP substrates were overlaid with a thin aqueous layer of DI water to preserve wet conditions.

### Bulk Refractive Index and Thermoplasmonic Detection

The localized surface plasmonic resonance (LSPR) phase sensing was based on a customized interferometric system driven by an LED-sourced white light sensing beam and analyzed by a spectrometer (AvaSpec, Avantes). The sensor chip was placed on a BK7 prism in the Kretschmann configuration to direct the incident light toward the dielectric interface at a nominal incident angle of 72°. A linear polarizer coupled with a birefringent crystal is employed to create a spectral interferogram that allows the application of windowed Fourier transform for real-time phase retrieval. Differential phase response to bulk refractive index (RI) variation was assessed by introducing NaCl solutions of various concentrations with RI ranging from 1.3333 to 1.3335.

A high-power 532 nm green laser (GML-FN-532 nm-1.5 W, CNI) was utilized to illuminate the AuNPs chip at a normal incident angle. An additional notch filter (NF533-17, Thorlabs) was employed to block the 532 nm light from entering the spectrometer. To calibrate the LSPR response with respect to temperature variations, the local surface temperature was measured using an infrared temperature sensor, and the phase responses at different plasmonic heated temperatures were recorded. The measurement was repeated three times to calculate the average *in situ* thermoplasmonics heating temperature.

### Surface Functionalization and Biomarker Detection by Hybridization

The surface of AuNPs within the microfluidic channel was functionalized via the creation of a self-assembled monolayer, through flushing ∼1 mL of 10 μM solutions containing freshly prepared oligonucleotide receptors at a flow rate 16 μL min^–1^ for a total duration of ∼60 min. Following the thiol-ligand exchange, the channel was rinsed with excess nuclease-free water for 10 min, which got the sensor ready for direct hybridization experiments. The sensing performance of AuNPs chips was examined by permeating target cDNA or the WHO standard SARS-CoV-2 of various concentrations, allowing for an exposure time of 900 s. Specifically, hybridization was allowed under the laser-driven plasmonic photothermal (PPT) heat (2.7 W cm^–2^) with ∼41 °C nominal temperature. The reference control was conducted using 0.1 wt % Bovine Serum Albumin (BSA, Bioss, bs-0292P) solution and unstimulated whole saliva (UWS) collected by passive drool. After rinsing with water and resting for 5 min, subjects allowed saliva to pool in the mouth and gently drooled into sterile tubes on ice for 3–5 min (≥0.5 mL). Samples were immediately capped, aliquoted, and stored at −80 °C.

### Viral Sequence Cyclic Cleavage Amplification

The thermoplastic-assisted dual-mode transducing detection was based on a combination of direct viral hybridization and amplification. Namely, the cyclic fluorescent probe cleavage was initiated after the direct viral sequence binding with V target 2. A 50 μL mixture of Endo-IV and 1 μM fluorescent probe (V probe 2) was injected within the microfluidic channel, and the binding-cleavage-dissociation process was activated using the homogenized 532 nm laser beam. The V probe 2, which is modified with a fluorophore-quencher pair on its 5′ (−ATTO532) and 3′ (−BHQ1) end, respectively, contains an abasic site toward its center acting as a substrate for Endonuclease IV. While the target-probe hybridized sequence is stable at temperatures below 50 °C, the cleavage of the abasic site under laser diode illumination marks the PPT dehybridization of double-stranded nucleic acid sequences, and activates the fluorophore. The response of fluorescence-induced phase change was recorded with the windowed Fourier transform calculation.

## Supplementary Material



## Data Availability

All data needed to evaluate the conclusions are present in the paper and/or the Supporting Information. Additional data related to this paper may be requested from the authors.

## Notes

The calculation for the ionic strength leading to polymer brush conformation changes, the calculation of the limit of quantification, the theoretical model for studying the LSPR near-fields, and the calculation of the LSPR enhancement factor. Additional figures include high-magnification SEM micrographs, quantitative binding site analysis, digital photographs of the AuNP arrays, environmental AFM images, persistence-diagram structure analysis, 2D small-angle X-ray scattering images, statistical dimension analysis of the block copolymer template and the AuNP spacing, transmittance spectra, phase response as a function of bulk RIU, phase response of AuNP arrays with short immersion time, *in situ* laser-induced local PPT heating characterization, FEM simulation plots, ESEF mapping, genomic information on the nucleic acids, receptor functionalization dynamics phase response, and full-scale LSPR responses of nucleic acid by direct hybridization and CFPC amplification (PDF)
