# Supplementary material for: Mismatch-Tuned Plasmonic Nanogap Networks on Block Copolymers for Ultrasensitive Nucleic Acid Quantification
Source: ACS Nanosci Au. 2026 Apr 14;6(4):570–81. doi: 10.1021/acsnanoscienceau.5c00190 (PMC13495505; doi:10.1021/acsnanoscienceau.5c00190)
Supplement: Supplementary file 1 [file ng5c00190_si_001.pdf]

Supplementary Materials for

# Mismatch-Tuned Plasmonic Nanogap Networks on Block Copolymers for Ultra-Sensitive Nucleic Acid Quantification

Yizhou Zhang[#], Ying Du[#], Martin Spillmann, Kazuto Akagi, Jiukai Tang, Shivaprakash N. Ramakrishna, Guangyu Qiu*, Jing Wang*

[#] These authors contributed equally to this work

*Corresponding author. Email: guangyuqiu@sjtu.edu.cn, jing.wang@ifu.baug.ethz.ch

### *Calculation for the Ionic Strength Leading to Collapsed Poly(4-Vinyl Pyridine), (P4VP)*

The interaction between citrate-acid functionalized gold nanoparticles (AuNPs) with P4VP entails a possible combination of electrostatic interaction, neutral hydrogen bonding, and ionic hydrogen bonding given by protonation of the pyridine moiety and the charged state of citric acid.[1] The extent of each is associated with solution pH. By estimating the local p$K_a$ ~ 5.23 similar to the bulk,[2] the number fraction of chemical species can be calculated, as

$$f_{C_7H_7NH^+} = \frac{[C_7H_7NH^+]}{[C_7H_7N] + [C_7H_7NH^+]} = \frac{1}{1 + 10^{pH-pK_a}} = 0.35$$

Additionally, while there are three different states of citric acid, the fraction of each citric acid moiety at a solution pH of 5.5 can be calculated based on the p$K_a$ values of each carboxylic acid at 3.13 (p$K_{a1}$), 4.76 (p$K_{a2}$), and 6.40 (p$K_{a3}$), respectively, as

$$\begin{aligned} f_{C_6H_8O_7} &= \frac{[C_6H_8O_7]}{[C_6H_8O_7] + [C_6H_7O_7{}^-] + [C_6H_6O_7{}^{2-}] + [C_6H_5O_7{}^{3-}]} \\ &= \frac{1}{1 + 10^{pH-pK_{a1}} + 10^{pH-pK_{a1}} \cdot 10^{pH-pK_{a2}} + 10^{pH-pK_{a1}} \cdot 10^{pH-pK_{a2}} \cdot 10^{pH-pK_{a3}}} \\ &= 5.9 \times 10^{-4} \end{aligned}$$

$$\begin{aligned} f_{C_6H_7O_7{}^-} &= \frac{[C_6H_7O_7{}^-]}{[C_6H_8O_7] + [C_6H_7O_7{}^-] + [C_6H_6O_7{}^{2-}] + [C_6H_5O_7{}^{3-}]} \\ &= \frac{1}{1 + (10^{pH-pK_{a1}})^{-1} + 10^{pH-pK_{a2}} + (10^{pH-pK_{a2}} \cdot 10^{pH-pK_{a3}})} = 0.14 \end{aligned}$$

$$f_{C_6H_6O_7^{2-}} = \frac{[C_6H_6O_7^{2-}]}{[C_6H_8O_7] + [C_6H_7O_7^{-}] + [C_6H_6O_7^{2-}] + [C_6H_5O_7^{3-}]}$$
$$= \frac{1}{1 + (10^{pH-pK_{a1}} \cdot 10^{pH-pK_{a2}})^{-1} + (10^{pH-pK_{a2}})^{-1} + 10^{pH-pK_{a3}}} = 0.76$$

$$f_{C_6H_5O_7^{3-}} = \frac{[C_6H_5O_7^{3-}]}{[C_6H_8O_7] + [C_6H_7O_7^{-}] + [C_6H_6O_7^{2-}] + [C_6H_5O_7^{3-}]}$$
$$= \frac{1}{1 + (10^{pH-pK_{a1}} \cdot 10^{pH-pK_{a2}} \cdot 10^{pH-pK_{a3}})^{-1} + (10^{pH-pK_{a2}} \cdot 10^{pH-pK_{a3}})^{-1} + (10^{pH-pK_{a3}})^{-1}}$$
$$= 0.096$$

Therefore, the total fraction of deprotonated carboxylic acid groups on the AuNPs can be estimated using the following expression, as

$$f_{COO^{-1}} = \frac{f_{C_6H_7O_7^{-}} + 2f_{C_6H_6O_7^{2-}} + 3f_{C_6H_5O_7^{3-}}}{3f_{C_6H_8O_7} + 3f_{C_6H_7O_7^{-}} + 3f_{C_6H_6O_7^{2-}} + 3f_{C_6H_5O_7^{3-}}} = 0.65$$

An estimation of P4VP chain length *l* can be based on assuming a moderate-density, nanoconfined polymer brush in good solvent, with a carbon-carbon bond length of 0.154 nm, which scales as $N^{0.8}$,[3] where *N* stands for the number of repeat units.

$$l = 1.54 \times N^{0.8} = 4.42\ nm$$

The above estimation suggests that the P4VP brush may extend into the column during the adsorption of AuNPs, and hence, the block copolymer template provides uniformly distributed anchoring sites of binding. Upon integration into microfluidic channels for sensing applications, the solution environment undergoes a transformation that reduces the fraction of charged P4VP moieties. This alteration can render the positioning of the attached AuNPs, potentially influencing the stability and uniformity of their spatial arrangement by transitioning to the salted brush regime at high ionic strength. The ionic strength, which causes brushes to collapse further, can be estimated as,

$$I = \frac{f_{C_7H_7NH^+} N\sigma}{RN_A}$$

Where σ is the estimated density of solvated brushes, R is the pore radius of the block copolymer at dry ~ 5 nm, and $N_A$ stands for Avogadro's number. At a neutral solution pH, the charged fraction reduces to ~ 1.7%, and the transitional ionic strength is calculated ~ 0.26 M.

***Calculation of the Limit of Quantification (LoQ)***

The lowest concentration at which the sensor can quantify the analyte is estimated from the average of the blank measures ($\bar{x}_B$) to ten times the standard deviation (σ).[4] Based on the calculated threshold response, the limit of quantification in 900 sec hybridization is determined to be:

$$LoQ = \bar{x}_B + 10\sigma = 0.53\, radian$$

Therefore, the corresponding LoQ of Target-1, with 67 base pairs, is determined to be 20 aM.

***Theoretical Models for Studying the Localized Surface Plasmon Resonance (LSPR) Near-Fields of AuNP Array***

The LSPR near-fields of AuNPs were simulated by the finite element method (FEM) based on the COMSOL software package (Burlington, MA, USA). In the three-dimensional (3D) LSPR model, AuNP array was lying on the glass substrate and surrounded by water. The laser was incident perpendicular to the water/AuNP/glass interface. For simplicity, it is assumed that the 4×6 array of AuNPs follow a close-packed hexagonal configuration, which locates on a glass substrate with cross section of 120 nm×150 nm. AuNPs were simplified to nanospheres with a diameter of 22.4 nm and were distributed with a gap of ~2.8 nm. To investigate the effects of the arrangement of AuNP on the electromagnetic (EM) field coupling strength, the EM distribution of 4×4 array of AuNPs in a quadrilateral configuration (the cross-sectional area of chip is 200 nm×200 nm) was also simulated for comparison. The Wave Optics Module was used to simulate the EM field profiles for the LSPR chip. Periodic boundary conditions were applied to represent the array structure. The source of electromagnetic radiation was a plane wave pulse with a wavelength of 532 nm at normal incidence. Based on the experimental condition, a linearly polarized transverse magnetic light arrived at the glass/water interface with the energy density of 2.8 W/m$^2$ and 1.5 W/m$^2$ for hexagonal and quadrilateral AuNP configuration, respectively. The glass substrate and water were considered as non-absorbing dielectrics whose refraction indices were real with n=1.330 for water, n=1.515 for the glass substrate. The other physical parameters of gold, glass and water were taken from the COMSOL library, and their dependence on temperature was not taken into account.

***Calculation of the LSPR enhancement factor***

The LSPR equivalent sensing enhancement factor (ESEF) of AuNPs was calculated using the following equation. The dimensionless enhancement factor was a normalized magnitude of hotspots that considered both the surface area and the EM amplitude corresponding to the total magnitude of the LSPR chip, and was divided by the reference magnitude of a bare glass without AuNPs.

$$ESEF = \frac{\frac{\oint E_{spot} \cdot ds}{\oint E \cdot ds}}{E_{substrate} \cdot S}$$

where $E_{spot}$ denotes the electric field amplitude inside hot spot and $E$ denotes the normal electric field intensity on the AuNPs located LSPR chip, while $E_{substrate}$ is the electric field intensity on the bare glass substrate, $S$ is the surface area of the bare glass substrate (120 nm×150 nm and 200 nm×200 nm for hexagonal and quadrilateral AuNP configuration, respectively.), and $s$ denotes the corresponding surface area for $E_{spot}$ and $E$ on AuNPs.

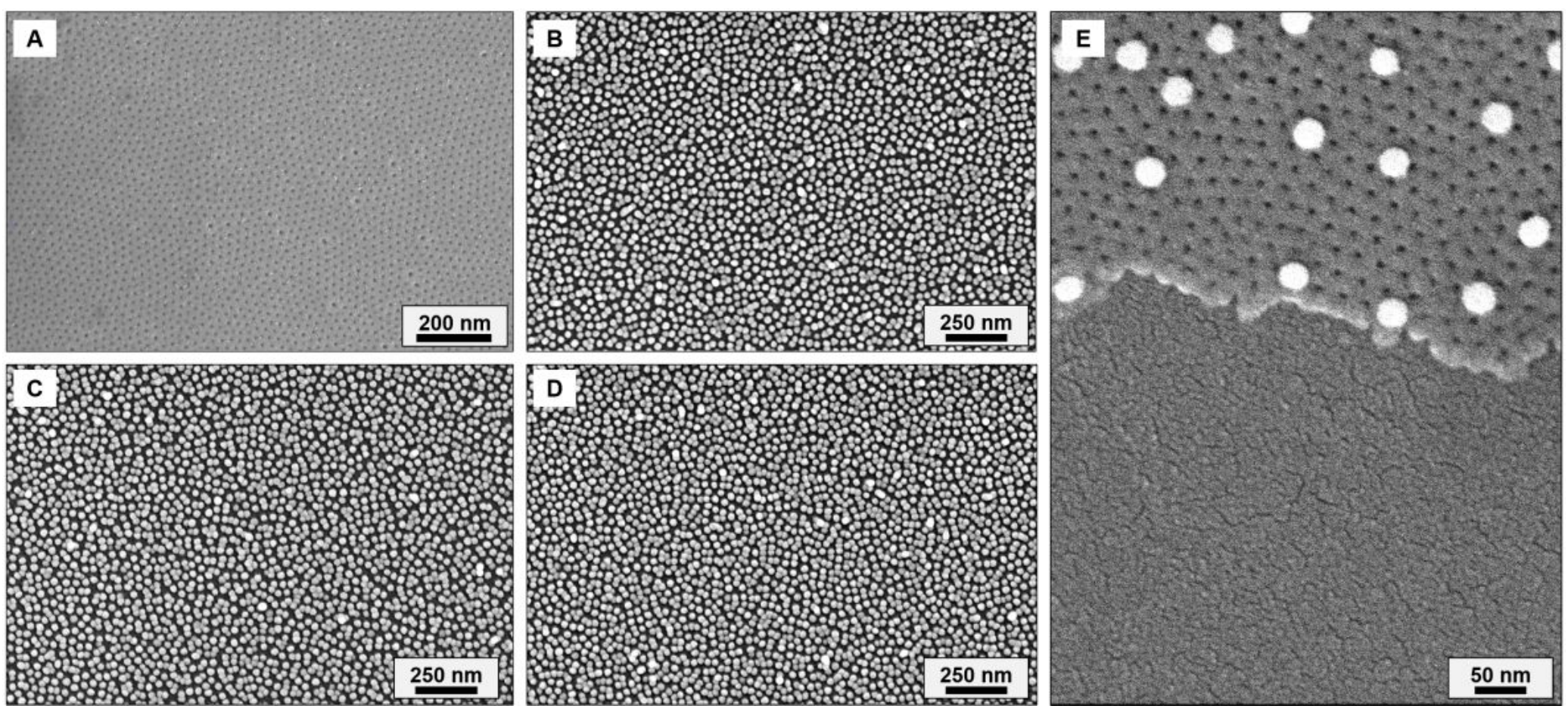


**Figure S1.** Scanning electron micrographs (SEM) reveal the progression of gold nanoparticle (AuNP) array formation on a self-assembled block copolymer template. Initially, (**A**) a thin film of the block copolymer was spin-coated onto a BK7 glass substrate. As the glass substrate was immersed in a solution containing 22.4 nm diameter AuNPs, the surface became populated with nanoparticles. Notably, the interstitial spaces between nanoparticles were filled over time, as evidenced by the changes from (**B**) 30 min of immersion to (**C**) 60 min, and eventually (**D**) 150 min post-immersion. In contrast, AuNPs with an average diameter of 28.2 nm are incompatible with the poly(4-vinylpyridine) spaces of $PS_{144}$-*b*-$P4VP_{66.5}$, as illustrated in (**E**). This SEM micrograph showcases the surface morphology on the edge of the coating, following a 5-minute immersion, highlighting the necessary match between the pore-to-pore distance and nanoparticle diameter for architecting confined spacings.

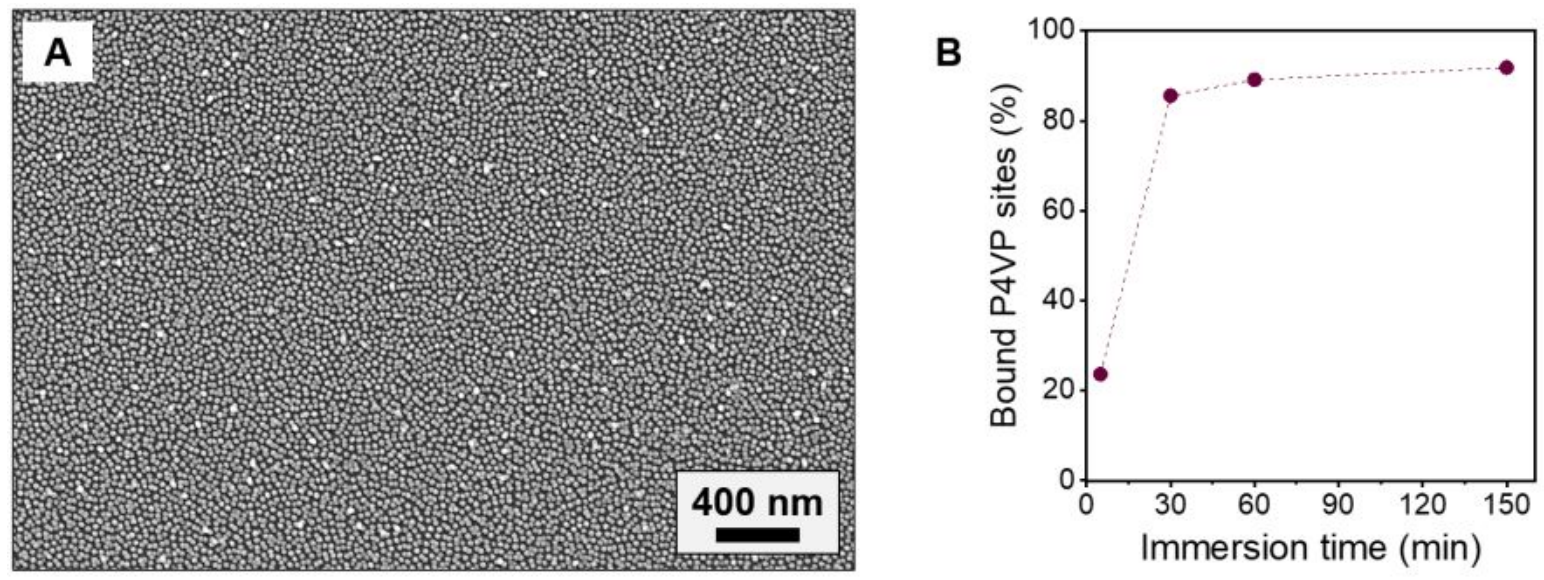


**Figure S2**. (**A**) Low-magnification SEM image showing uniform adsorption of AuNPs across the block copolymer template 150 min post-immersion. (**B**) Quantitative image analysis demonstrates time-dependent surface binding site saturation, with more than 90% of P4VP domains occupied after 150 min of immersion.

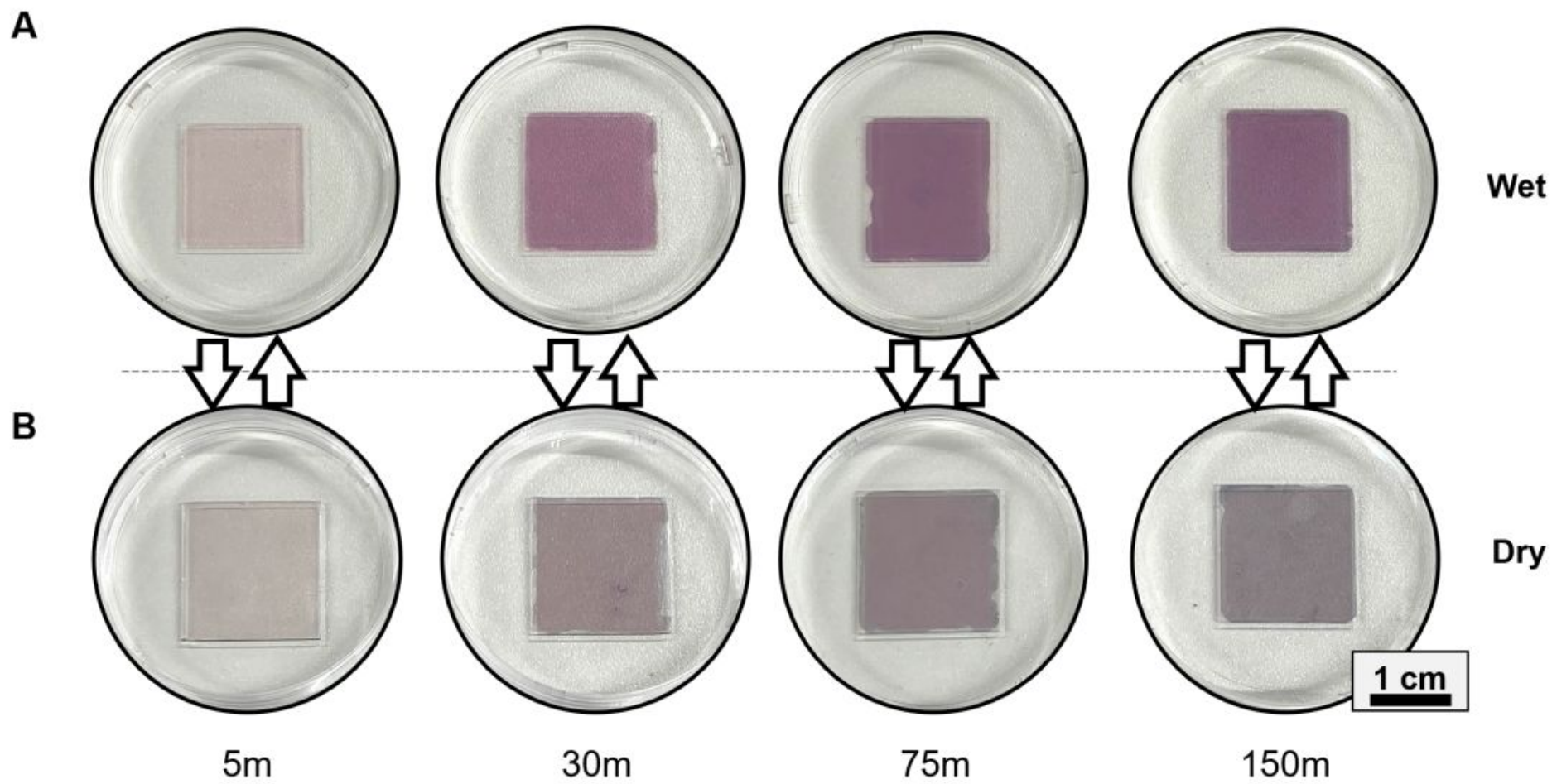


**Figure S3**. Variation in the reflectance of the AuNP array with changes in the environmental dielectric constant. The reflectance properties of the AuNP array are influenced by alterations in the surrounding dielectric constant, evidenced by a visible color shift when the glass substrate is transferred from (**A**) a deionized water bath to (**B**) a dry air environment. The collapse of the poly(4-vinyl pyridine) polymer brushes in a dry environment leads to the aggregation of gold nanoparticles, resulting in a noticeable color transition of the chip upon visual inspection, from purple in a wet state to plum slate once air-dried. This wetting-drying process is fully reversible.

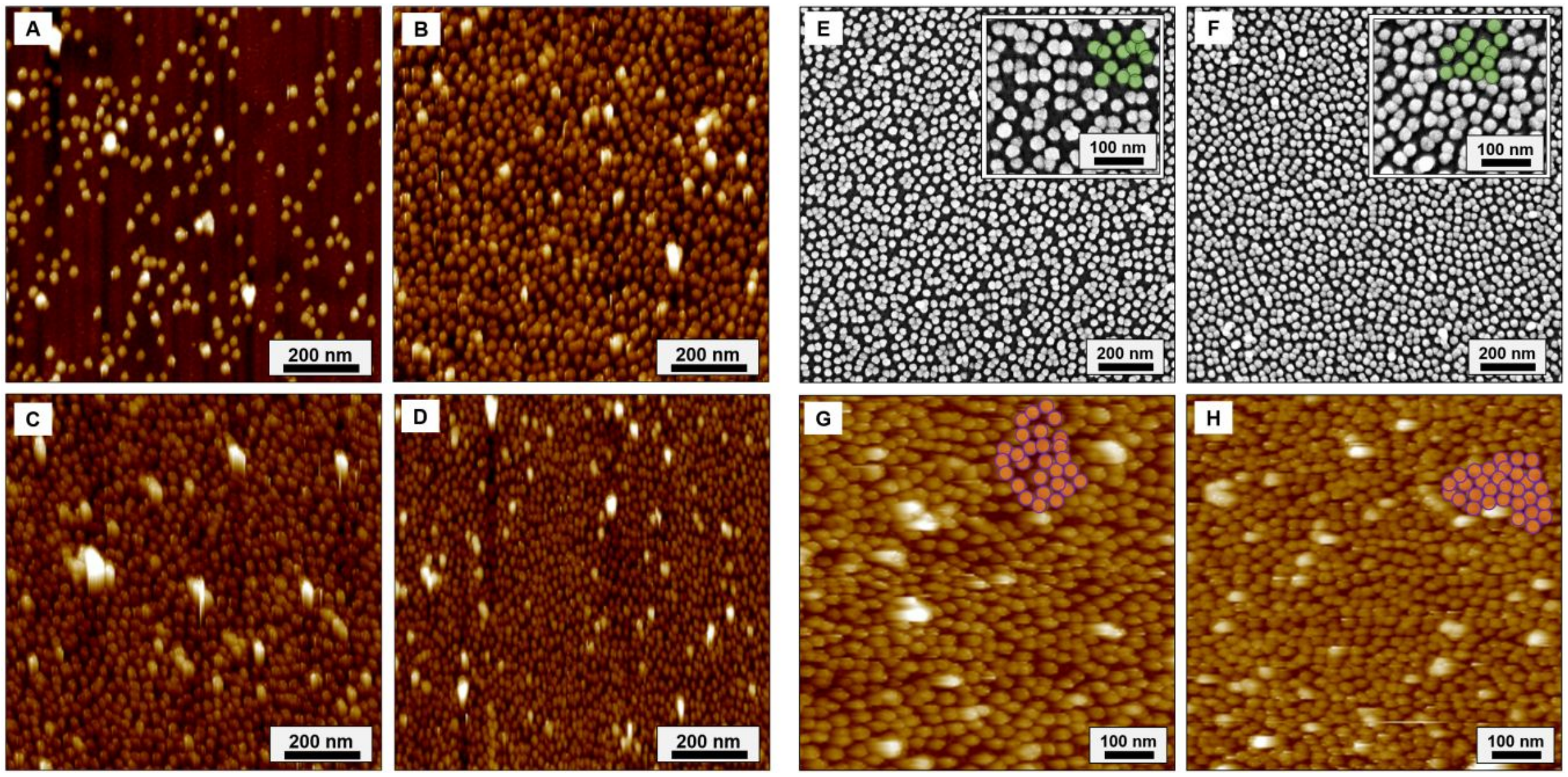


**Figure S4.** The time-dependent evolution of the AuNP array was analyzed using SEM and atomic force microscopy (AFM). AFM images taken in water demonstrate that the particle density within the AuNP array increases by immersing the block copolymer thin film in AuNP solution from 5 min (**A**) to 30 min (**B**), and then 75 min (**C**) to 150 min (**D**). At a shorter exposure time of 30 min, AuNPs are more sparsely distributed across the surface, resulting in a lack of effective coupling, as demonstrated in the SEM micrograph in (**E**), taken *in vacuuo*, and the magnified AFM image in (**G**), captured in an aqueous environment. When the exposure time is extended to 150 min, there is a notable increase in the occupancy of binding sites, leading to a more closely packed arrangement of AuNPs, shown in SEM (**F**) and AFM (**H**) images, indicating more efficient nanoparticle packing for enhanced coupling.

**A**

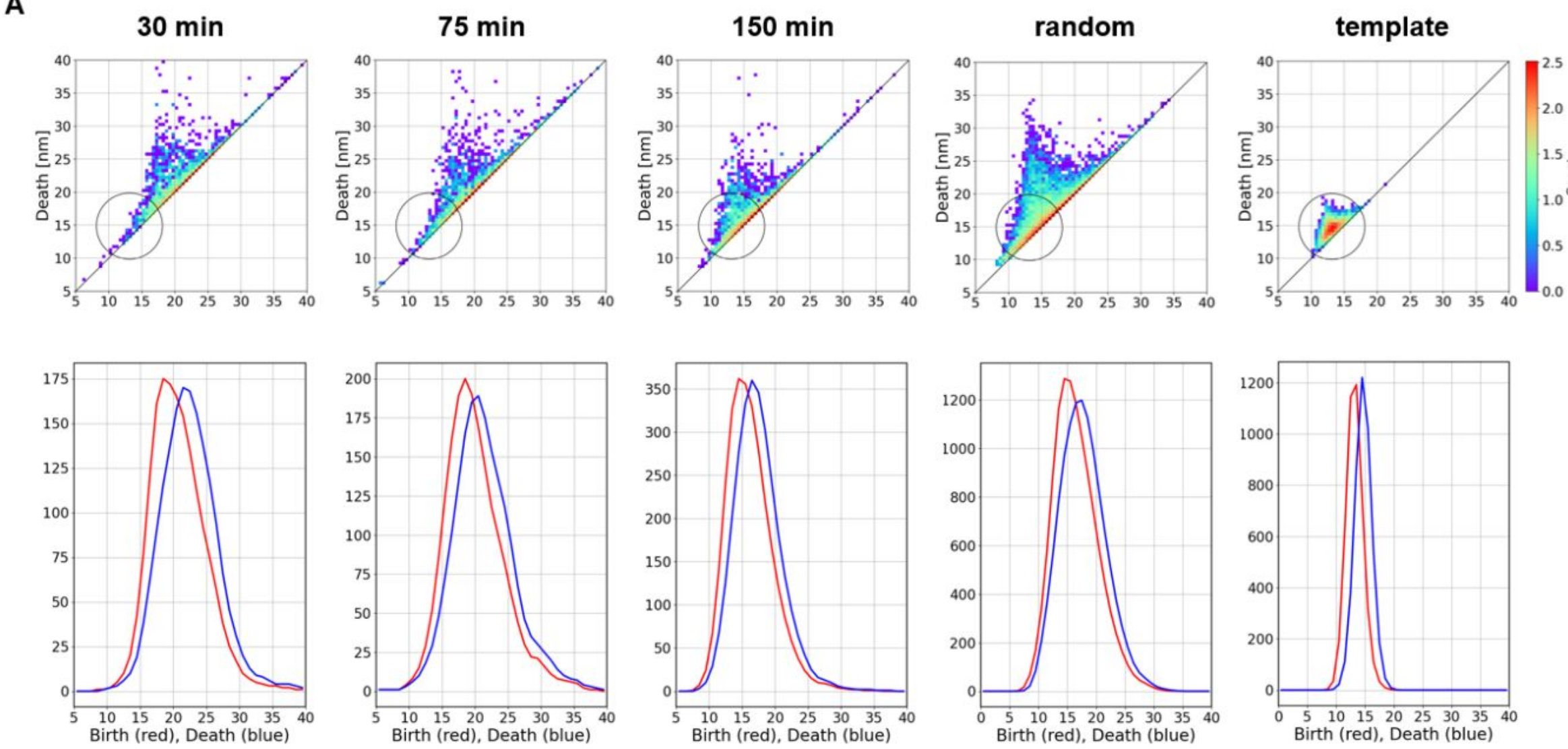


**B**

| Sample name | | 30 min | 75 min | 150 min | random | template |
|---|---|---|---|---|---|---|
| Birth (nm) | mode | 18.9 | 17.3 | 14.3 | 13.9 | 13.1 |
| | stdev | 4.1 | 4.9 | 3.4 | 3.7 | 1.1 |
| Death (nm) | mode | 22.3 | 19.7 | 16.7 | 18.0 | 14.9 |
| | stdev | 4.2 | 5.1 | 3.5 | 3.8 | 1.0 |

**Figure S5.** (**A**) "30min", "75min", and "150min": Persistence Diagrams (PD) corresponding to the ring structures formed by the arrangement of particles observed by water environment AFM. The horizontal axis represents the birth values, the vertical axis represents the death values, and the color indicates the density of birth-death pairs. "random": A PD corresponding to the ring structures formed by randomly arranged 15 nm-diameter disks (non-overlapping), with the same density as "150min". "template": A PD corresponding to the ring structures formed by the arrangement of pits on the substrate observed by SEM. The circles drawn on each PD represent, for comparison, the distribution range of birth-death pairs in the PD of "template". In the PD of the "template", where pits are arranged in a regular pattern, the birth-death pairs are densely concentrated within a narrow region corresponding to equilateral triangles. The PDs of "30min" and "75min" show broader distributions, with fewer birth-death pairs within the circled area. The PD of "150min" contains many birth-death pairs within the circled area. Although "random" also has many birth-death pairs within the circled area, its PD exhibits sharp features resulting from the randomness of the arrangement. (**B**) A table summarizing the mode and standard deviation of the birth and death value histograms. In "30min" and "75min", both the modes and standard deviations of the birth and death values are larger than those in the "template", indicating that the particle arrangement is sparse and random. In "150min", the modes of the birth and death values are close to those of the "template". Considering that the birth value corresponds to the longest edge of a triangle and the standard deviation is slightly larger, this suggests an arrangement of nearly equilateral triangles of similar size to the "template" but with slight distortion. In "random", the mode of the birth values is even closer to that of the "template", but both the mode and standard deviation of the death values are larger than those in "150min". This implies the presence of a larger number of polygons other than triangles, again suggesting that the arrangement in "150min" is closer to a triangular lattice.

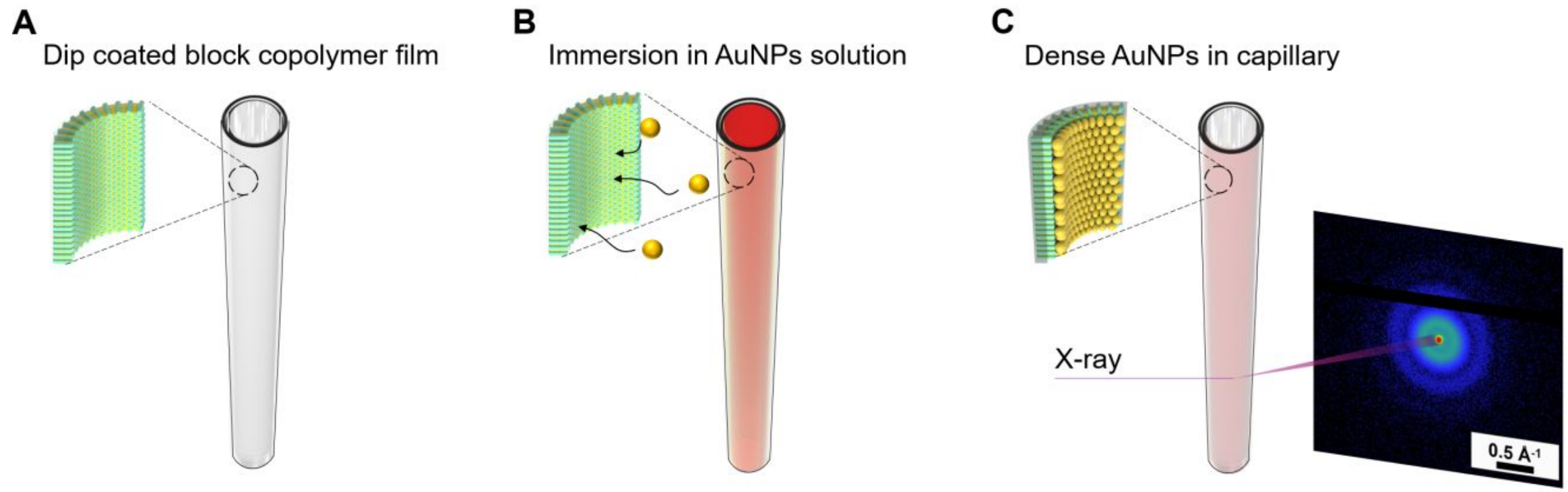


**Figure S6.** Schematic for the preparation process of the AuNPs array for small-angle X-ray scattering (SAXS) analysis. (**A**) A capillary tube was dipped into a 0.5 wt% $PS_{144}$-*b*-$P4VP_{66.5}$ solution in toluene. The solvent was allowed to fully evaporate, leaving a polymer thin film coating the interior of the capillary, which was then immersed in a bath of ethanol for surface reconstruction. (**B**) Subsequently, the capillary tube with an interior coated block copolymer was submerged within a bath of AuNPs solution for 150 min, and rinsed with excess 18 MΩ deionized (DI) water. (**C**) For SAXS characterization, the capillary was filled with DI water and sealed at both ends with epoxy. The X-ray was directed across the diameter of the capillary.

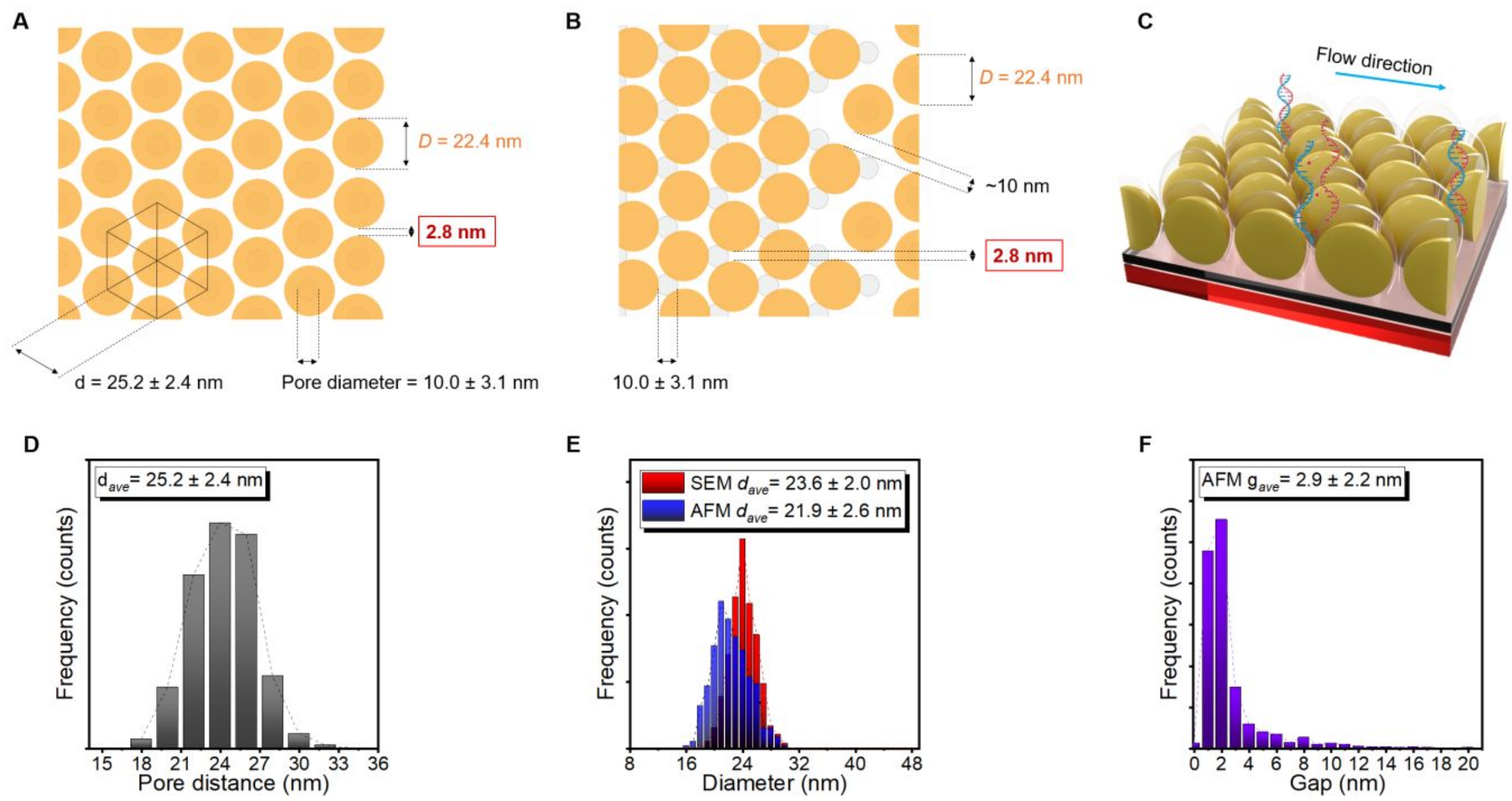


**Figure S7.** Schematic and statistical analysis of the self-assembled block copolymer template and the calculated AuNPs spacing. (**A**) Top-down geometrical view of 22.4 nm AuNPs anchored on a self-assembled block copolymer, with a center-to-center spacing of 25.2 nm. In this case, an average confined spacing of 2.8 nm is assumed under homogeneous nanoparticle distribution. (**B**) Illustration of the potential binding mismatch when AuNPs are anchored between three adjacent poly (4-vinyl pyridine) domains, resulting in a large gap of ~10 nm, too small for the accommodation of additional AuNPs. (**C**) Perspective view of nucleic acids adsorbed within 2.8 nm gaps in a microfluidic channel with homogeneous AuNPs distribution. (**D**) Statistical distribution of center-to-center distances of the block copolymer measured from SEM images. (**E**) Average size distribution of AuNPs as measured by SEM *in vacuuo* and AFM in an aqueous environment, which is consistent with the SAXS experiment. (**F**) The statistical distribution of gap sizes in the aqueous environment was determined from AFM micrographs, highlighting the precision in targeting an average confined spacing below 3 nm. 85% of gaps range from 1 to 4 nm.

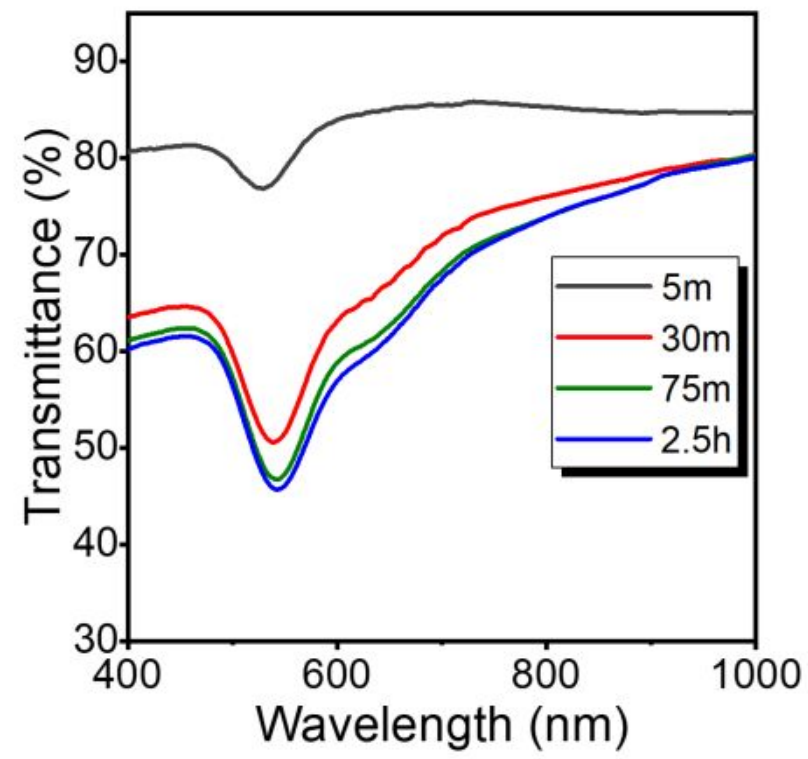


**Figure S8.** Transmittance spectra of the AuNPs array as a function of development time. The emergence of enhanced coupling at ~630 nm indicates the formation of closely packed AuNPs with increased solution exposure time.

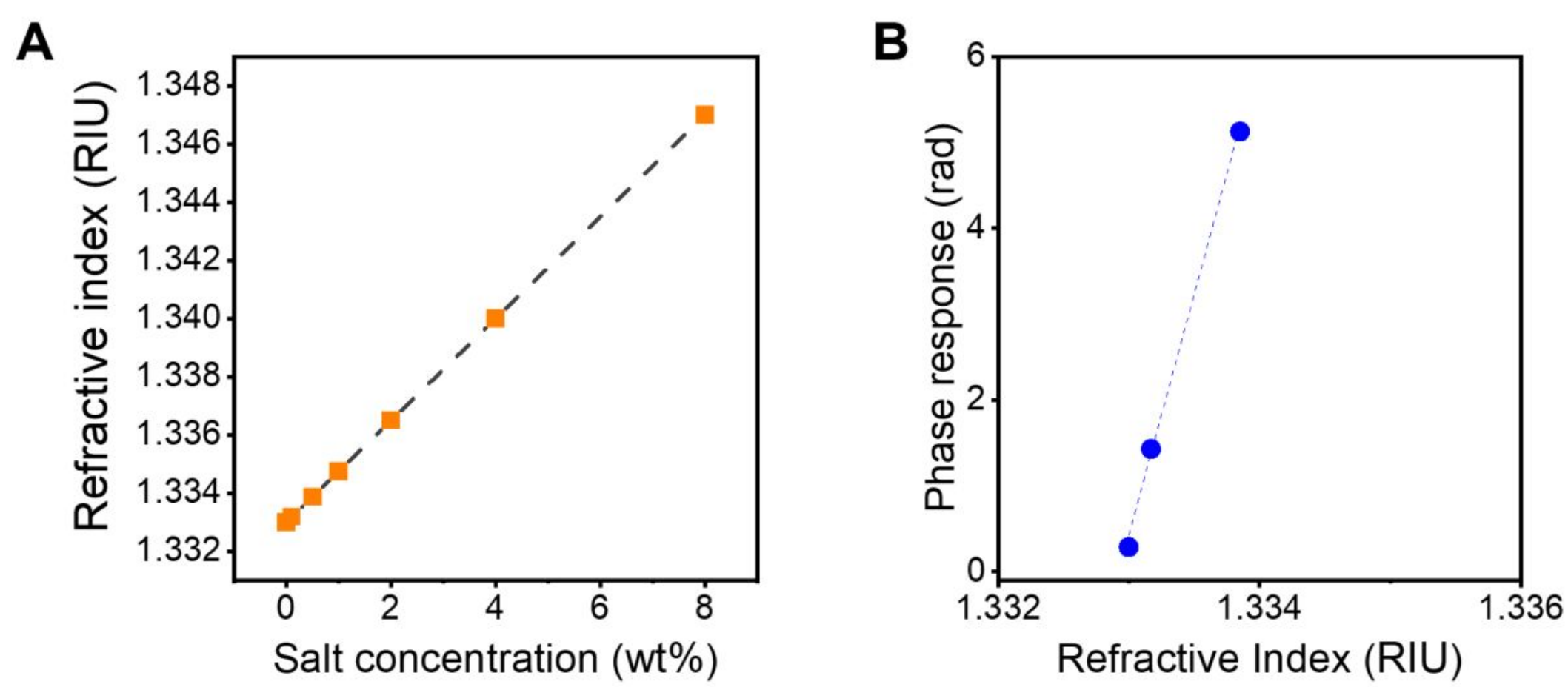


**Figure S9.** (**A**) Bulk RIU plotted as a function of NaCl solution concentrations. (**B**) Phase response recorded with alternating NaCl concentration, and hence, RIU ranging from ~ 0.1333 to 0.1334. The calculated bulk RIS is ~ 5600 rad $RIU^{-1}$ at low salt concentrations.

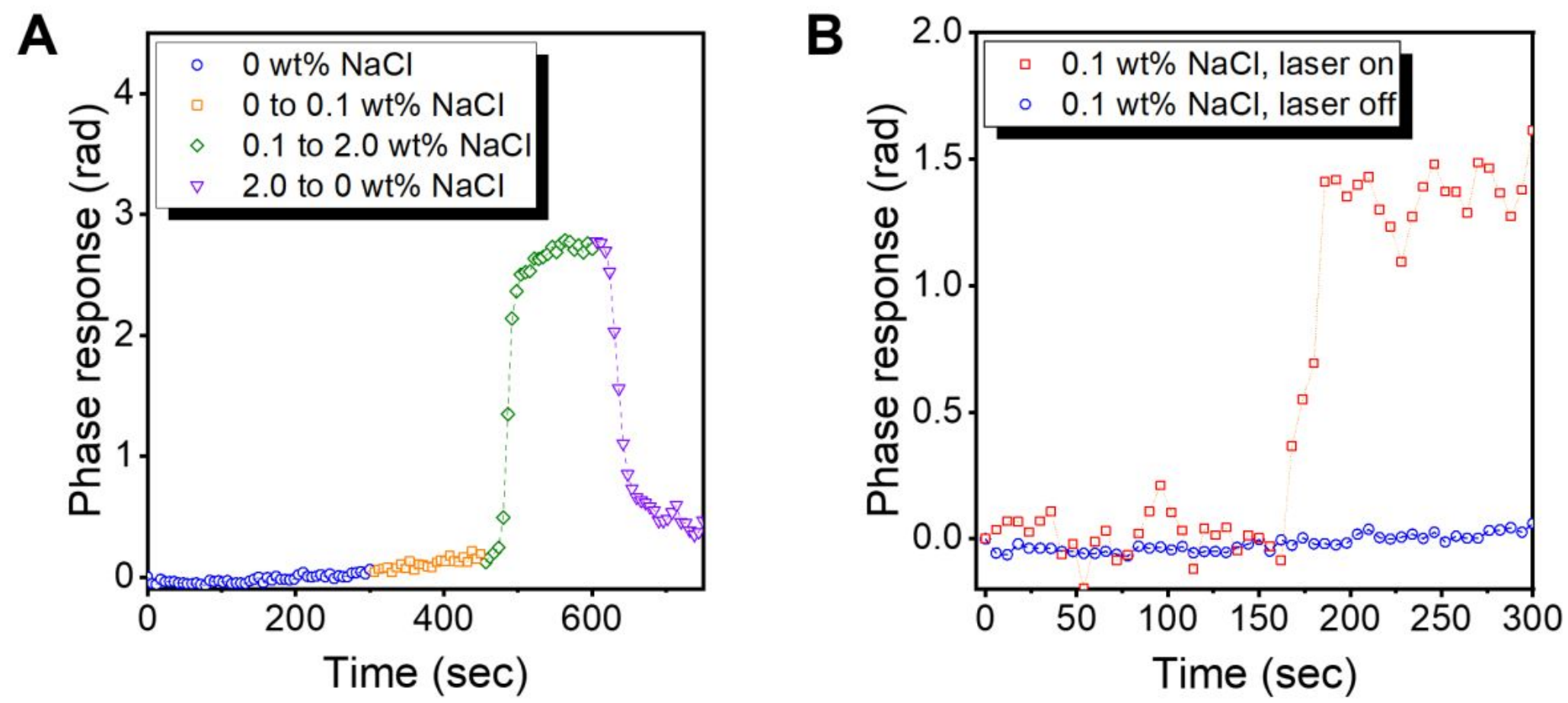


**Figure S10.** Phase response of AuNP prepared with 30-minute immersion time. (**A**) Real-time phase shift with increasing NaCl concentration from 0 to 2 wt%. Unlike the 150-minute AuNP, the chip shows negligible response to 0.1 wt% NaCl. (**B**) The excitation by the 532 nm laser under normal incidence enhances the phase response of 0.1 wt% NaCl, with a RIS of 8300 rad $RIU^{-1}$.

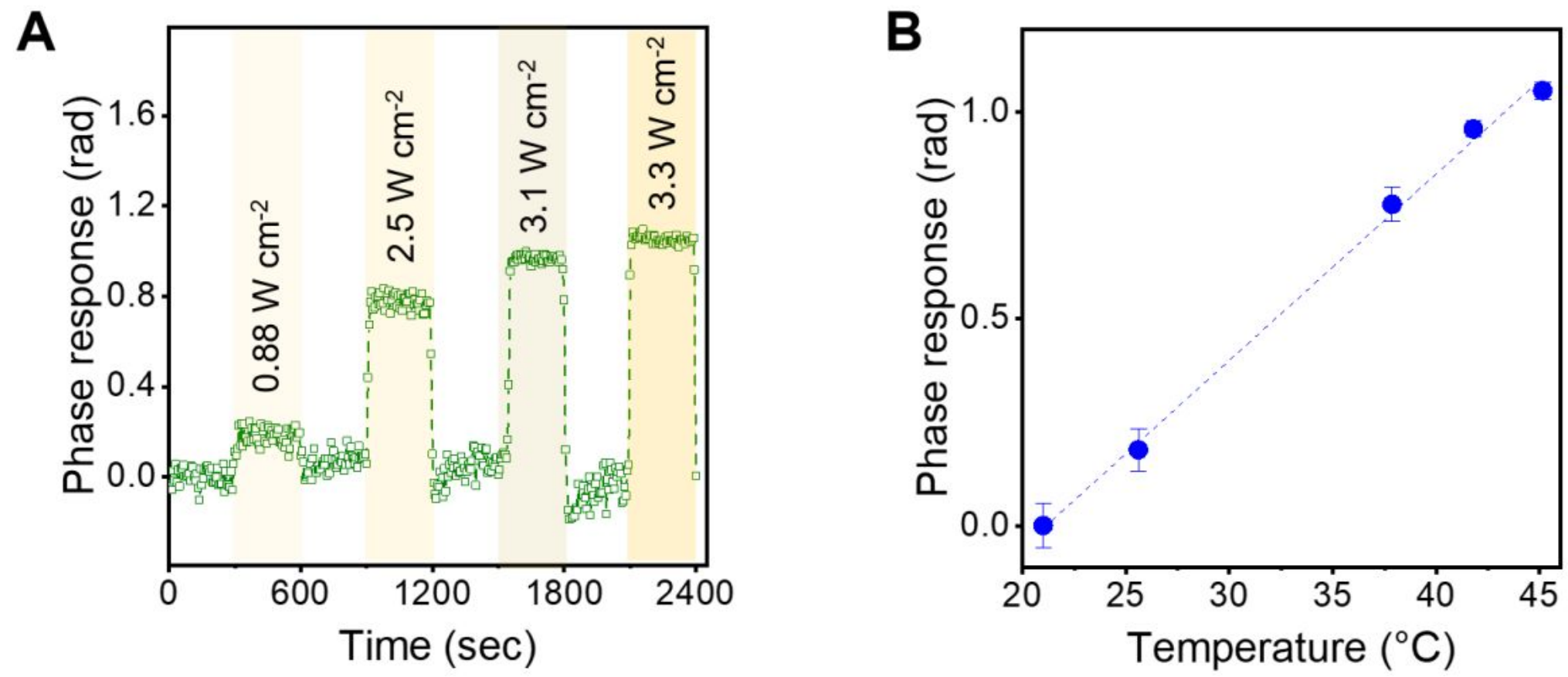


**Figure S11.** In-situ characterization of laser-induced local PPT heating on AuNPs surface at varying input powers. (**A**) Temporal PPT-induced plasmonic phase shift recorded as a function of laser excitation across a power range from 0.88 to 3.3 W $cm^{-2}$. (**B**) Variation in phase responses correlated with the local surface temperature of templated AuNPs.

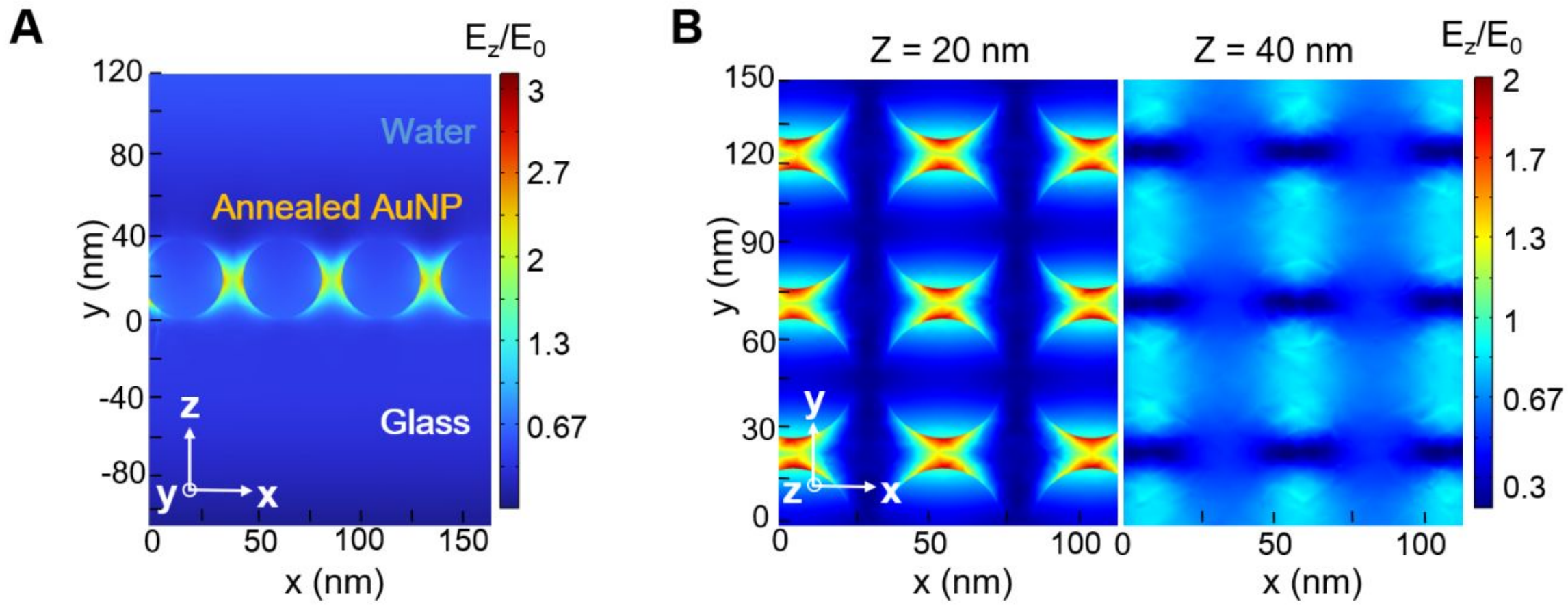


**Figure S12.** FEM simulation of the local electric field for AuNP array with quadrilateral configuration, analogous to the design of previously reported thermally annealed sensor chips. The mapping of near-field intensities is demonstrated in (A) side-view perspective at the glass/AuNP/water interface, where the maximum field intensity reaches $6 \times 10^8$ V m$^{-1}$ and the EM magnitude is $3 \times 10^8$ V m$^{-1}$ for bare glass. The top view cross-sections at (B) z = 20 nm and z = 40 nm indicate the location of the maximum electric field, which contributes a maximum $E/E_0 \sim 2$ and 1.2, respectively.

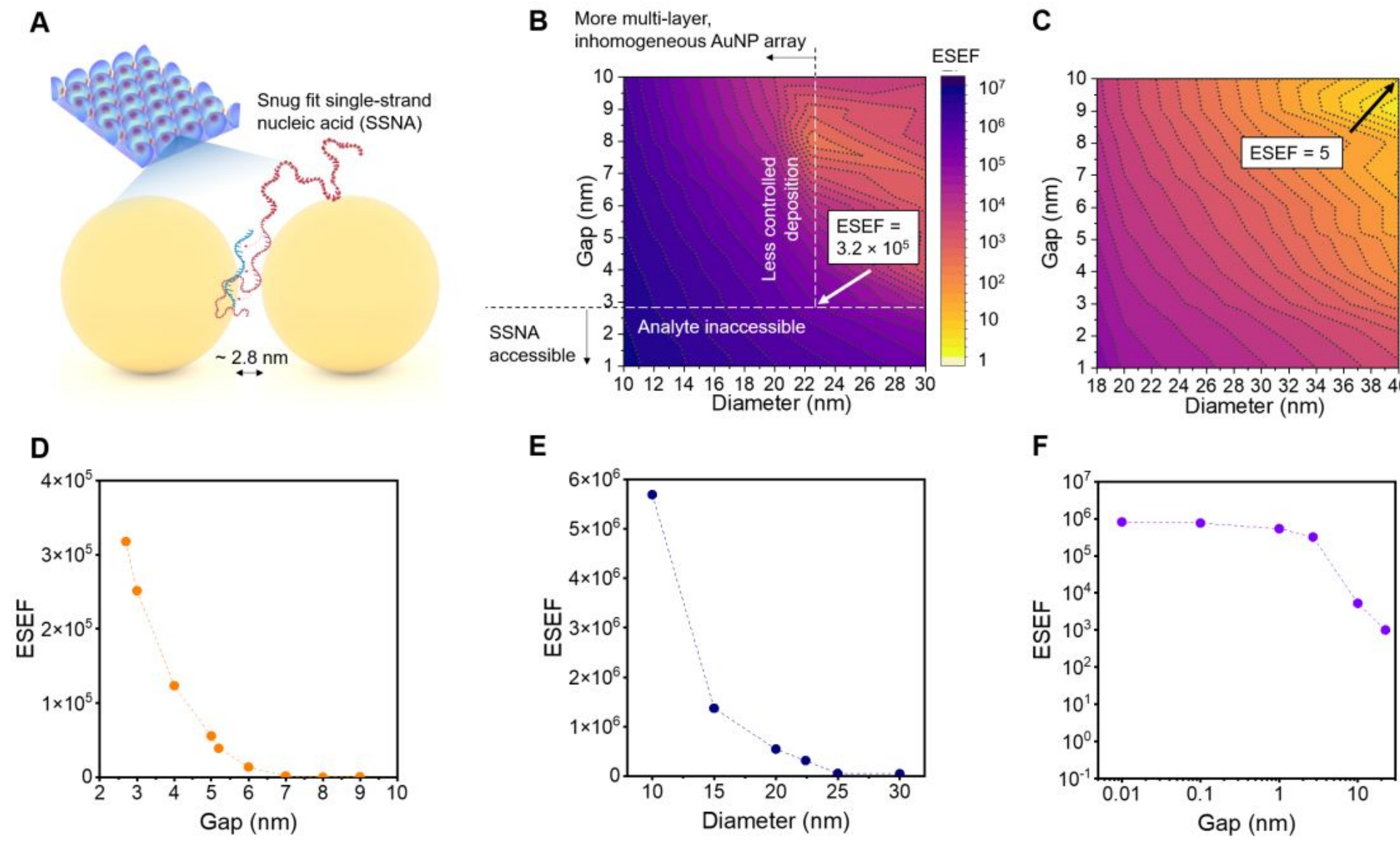


**Figure S13.** (A) Schematic of the close-packed AuNPs confining an interstitial spacing of ~ 2.8 nm, sufficient to accommodate single-strand nucleic acids in the sensing process. (B) At the hexagonally arranged configuration with 22.4 nm AuNP and 2.8 nm spacing, the integration of electromagnetic field with respect to surface area contributes an equivalent sensing enhancement factor (ESEF) ~ 3.2 × $10^5$. For reference, adsorption at lower solution pH, or with small size AuNP (i.e., < 10 nm) is ill-suited because aligning nanoparticles in-plane with controllable interparticle distances presents challenges. Small nanoparticles may be adsorbed into polymer brushes, instead of atop the brush, enabling LSPR to occur at multi-scale heterogeneous spacings and contribute to an inhomogeneous distribution of the electric field. (C) The integration, assuming a quadrilateral configuration with 40 nm AuNP and an intraparticle distance of 10 nm contributes a reduced ESEF ~ 5. Extrapolated results from the ESEF mapping show that (D) varying the gap distance at a fixed AuNP diameter, and (E) varying the AuNP diameter at a constant gap distance have a significant impact on the calculated enhancement factor, although smaller AuNPs may increase the likelihood of aggregation (*e.g.*, multiple particles occupying a single adsorption site). (F) Enlarging the AuNP diameter while maintaining a constant template domain spacing leads to substantial ESEF enhancement, particularly for small particles with gap distances larger than ~5 nm. However, a perfectly dimensionally matched configuration with very small spacing provides only marginal gains when the gap distance is reduced below ~3 nm.

A

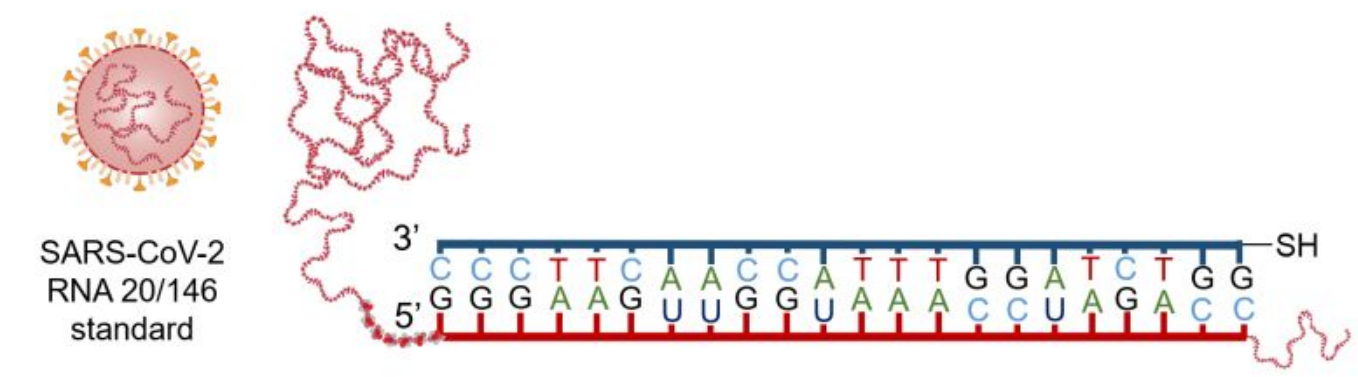


B

| Name | Sequence (5'-3') | Length |
| --- | --- | --- |
| Receptor-1 | THIOL-G GTC TAG GTT TAC CAA CTT CCC | 21 |
| Target-1 | CTA CTG TAC GTG AAG TGC TGT CTG ACA GAG AAT TAC ATC TTT CAT GGG AAG TTG GTA AAC CTA GAC C | 67 |
| WHO target | WHO International Standard for SARS-CoV-2 RNA 20/146 | |
| Receptor-2 | THIOL-GGA TTG CGG GTG CCA ATG TGA | 21 |
| Target-2 | GGG AGC CTT GAA TAC ACC AAA AGA TCA CAT TGG CAC CCG CAA TCC | 45 |
| Probe-2 | ATTO532-C GGG TTT GGT GTA T(**AP**)C AAG GCT CCC G-BHQ1 | 45 |

**Figure S14.** Genomic information of the nucleic acid receptors and targets employed for SARS-CoV-2 detection on the AuNPs surface. Two synthetic viral sequences encoding the part of the ORF-1ab region were selected as Target-1 and Target-2, which are matched with their target using the basic local alignment search tool (BLAST). The full genome target includes the first WHO international Standard for SARS-CoV-2 RNA for Nucleic acid Amplification Technique (NAT)-based assays consisting of acid-heat inactivated England/02/2020 SARS-CoV-2 isolate.

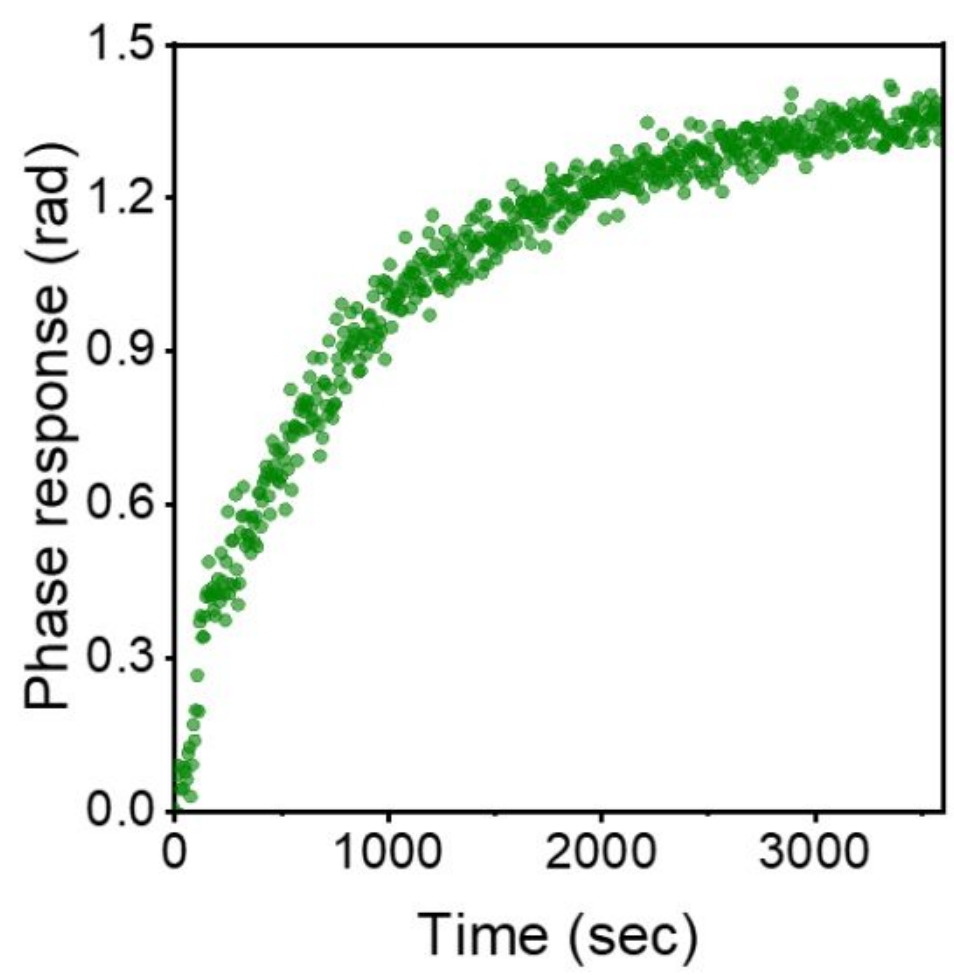


**Figure S15.** Exemplary functionalization dynamics based on the reaction between templated AuNPs surface and Receptor-1thiol-cDNA ligands. The phase response was recorded for ~3600s. The functionalization process involved flowing a viral sequence receptor at a concentration of 10 µM across the surface at a flow rate of 16 µL $min^{-1}$ for 60 min.

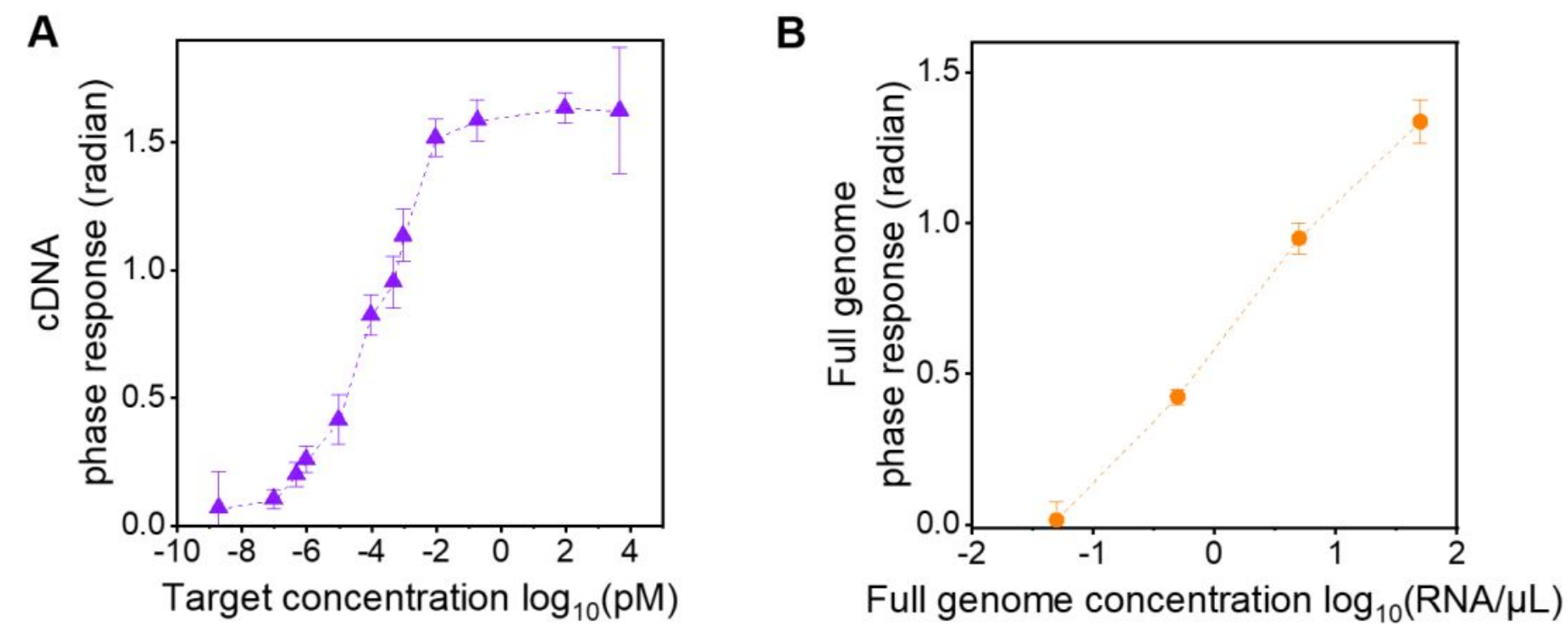


**Figure S16.** Full-scale LSPR response for model nucleic acid sequences. (A) The Target-1 ORF-1ab bioassay exhibits a significant response until the target concentration falls below ~ 0.1 aM. The saturation concentration of the response occurs at ~ 0.01 pM. (B) The response of the WHO international standard SARS-CoV-2 RNA (20/146), with readable responses until the genome concentration decreases to below ~ 0.1 RNA µL$^{-1}$.

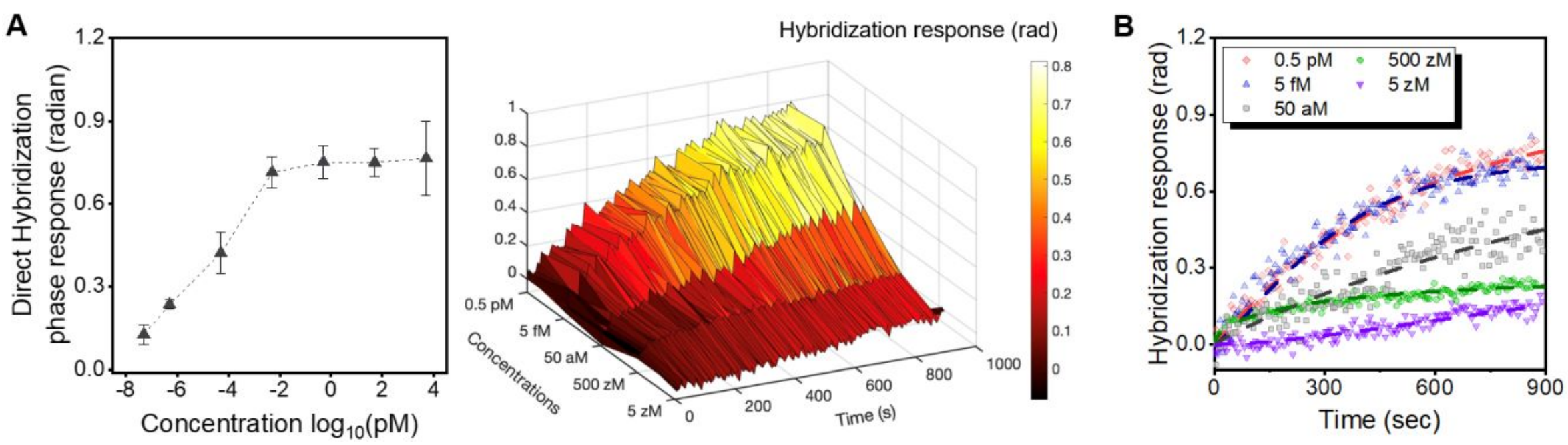


**Figure S17.** Full-scale LSPR readout of the direct binding mode sensing pathway. (A) The direct binding mode for Target-2 exhibits a detection range similar to that of Target-1, with readable responses from approximately 0.1 aM to saturation at ~ 0.01 pM. A reduced response, in comparison with NV target 1, may originate from a lower molecular weight of the target. (B) Real-time response data for detecting the Target-2 sequence (comprising 45 nucleotides) within the initial 900 seconds across a concentration range from 5 zM to 0.5 pM.

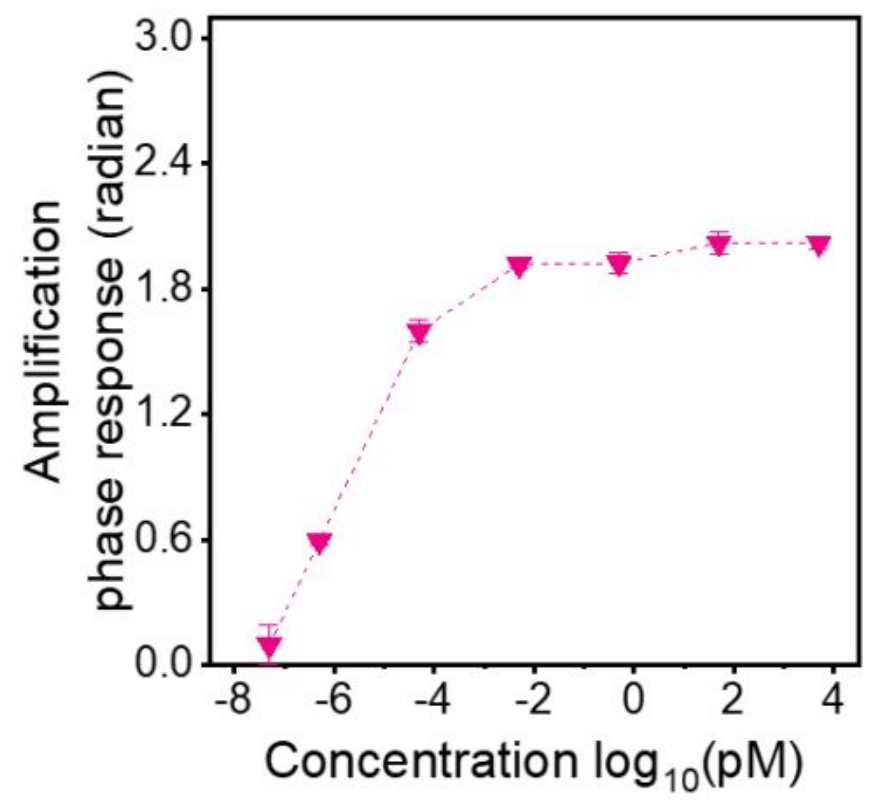


**Figure S18.** The CFPC amplification-augmented bioassay readout after 30 minutes exhibits an enhanced response and detection range from approximately 0.1 aM to about 1 fM.

**Table S1.** Amplification-based approaches for SARS-CoV-2 detection

| Amplification-based Methods | | | | | |
|---|---|---|---|---|---|
| **Assay Name** | **Principle** | **Target Gene** | **Assay Time (min)** | **Limit of Detection (LOD) or Otherwise Defined** | **Ref.** |
| CDC COVID-19 RT-PCR panel assay | RT-PCR | N | 44 | 1.2 copies $\mu L^{-1}$ | [5] |
| RT-LAMP assay | RT-LAMP | N | 30 | 100 copies per reaction | [6] |
| mRT-LAMP-LFB | RT-LAMP with lateral flow biosensor | N, ORF1ab | 40 | 12 copies per reaction | [7] |
| COVID-19-LAMP | RT-LAMP with colorimetric assay | E, OR3a | 60 | 42 copies per reaction | [8] |
| Suitcase Lab | RT-RPA | E, N | 15 | 15 copies per reaction | [9] |
| Exo-IQ | RT-RPA | N | 7 (first result) | ~7.7 copies per reaction | [10] |
| SARS-CoV-2 DETECTR Reagent Kit | RT-LAMP, CRISPR-Cas12 | E, N | 45 | 10 copies $\mu L^{-1}$ | [11] |

**Table S2.** Biosensor-based approaches for SARS-CoV-2 detection

| Sensors | | | | | |
|---|---|---|---|---|---|
| **Sensing Mechanism** | **Sensing Element** | **Target** | **Time (min)** | **Limit of Detection (LOD) or Otherwise Defined** | **Ref.** |
| Electrochemical | Graphene-based SPE with monoclonal antispike antibody | Spike protein | 45 | 20 μg $mL^{-1}$<br>$5.5 \times 10^{5}$ PFU/mL | [12] |
| Electrochemical | Antibodies immobilized on magnetic beads | Spike protein or Nucleocapsid protein | 30 | 19 ng $mL^{-1}$ or 8 ng $mL^{-1}$ | [13] |
| Electrochemical | Hybridization of the rolling circle amplification amplicons with redox-active labelled probes | S gene or N gene | < 120 | 1 copy $\mu L^{-1}$ | [14] |
| Electrochemical | p-sulfocalix[8]arene functionalized graphene | SARS-CoV-2 RNA | < 0.2 | 0.2 copy $\mu L^{-1}$ | [15] |
| Electrochemical | $Cu_2O$ nanocubes coated with IgG spike antibody | Spike protein | < 20 | 0.04 fg $mL^{-1}$ | [16] |
| Electrochemical | Single-walled carbon nanotube with redox-tagged DNA aptamer | Spike protein | 120 | 7 nM | [17] |
| Electrochemical | Carbon nanofiber functionalized with nucleocapsid antigen | Nucleocapsid protein | 20 | 0.04 fg $mL^{-1}$ | [18] |
| FET | Single-walled carbon nanotube functionalized with antibody | Spike protein or nucleocapsid protein | < 5 | 19 ng $mL^{-1}$ or<br>8 ng $mL^{-1}$ | [19] |
| FET | Graphene functionalized with antibody | Antigen protein or cultured SARS-CoV-2 RNA | > 1 | 0.24 copy $\mu L^{-1}$ | [20] |
| FET | AuNP-decorated with phosphorodiamidate morpholino oligos | SARS-CoV-2 RNA | 2 | 0.37 fM | [21] |
| SERS | Spike protein DNA aptamer on Au Nanopopcorn surface | Spike protein | 15 | 10 copies $\mu L^{-1}$ | [22] |
| SERS | Bumpy Core-Shell NP with detection antibody | Spike protein | 120 + 180 | 710 aM | [23] |
| SERS | Single-chain Fv recombinant antibody on magnetic nanoparticle | Spike protein | 30 | 257 fg $mL^{-1}$ | [24] |
| Colorimetric | Spike and nucleocapsid gold colloid assay | Anti-SARS-CoV-2 IgM/ IgG | 20 | 1 ng $mL^{-1}$ | [25] |
| Colorimetric | Selenium nanoparticle-modified SARS-CoV-2 nucleoprotein | Anti-SARS-CoV-2 IgM/ IgG | 5 | 20 ng $mL^{-1}$ (IgM)<br>5 ng $mL^{-1}$ (IgG) | [26] |
| Colorimetric | AuNPs with oligonucleotide probes | Oligonucleotides | 5 | 5 pM | [27] |
| Colorimetric | ASO capped AuNPs | N Gene | 10 | 0.18 ng $\mu L^{-1}$ | [28] |
| LSPR | Au Nanorods with recombinant N protein | Nucleocapsid protein | 10 | 1 pM | [29] |
| LSPR | AuNPs with nanobodies (NB1D6 and NB4E9) | Spike receptor-binding domain | 30 | 0.01 ng $mL^{-1}$ | [30] |
| LSPR | Gold nanoislands with complementary DNA receptor | Oligonucleotides | 20 | 0.22 pM | [31] |
| LSPR | Gold nanoislands with amplification-based cyclic fluorescence probe cleavage | Oligonucleotides | 20 | 0.28 fM | [32], [33] |
| LSPR | AuNP hotspot array with complementary DNA receptor | Full SARS-CoV-2 genome | 30 | 0.88 copy $\mu L^{-1}$<br>(Limit of quantification) | This work |
| LSPR | AuNP hotspot array with amplification-based cyclic fluorescence probe cleavage | Oligonucleotides | < 6 | 0.12 aM<br>(Limit of quantification) | This work |

## References


**(1)** Kim, Y. C.; Composto, R. J.; Winey, K. I. pH-Mediated Size-Selective Adsorption of Gold Nanoparticles on Diblock Copolymer Brushes. *ACS Nano* **2023**, *17*, 9224–9234.

**(2)** Kennemur, J. G. Poly(vinylpyridine) Segments in Block Copolymers: Synthesis, Self-Assembly, and Versatility. *Macromolecules* **2019**, *52*, 1354–1370.

**(3)** Hamilton, W.; Smith, G.; Alcantar, N.; Majewski, J.; Toomey, R.; Kuhl, T. Determining the Density Profile of Confined Polymer Brushes with Neutron Reflectivity. *J. Polym. Sci., Part B: Polym. Phys.* **2004**, *42*, 3290–3301.

**(4)** Long, G. L.; Winefordner, J. D. Limit of Detection. A Closer Look at the IUPAC Definition. *Anal. Chem.* **1983**, *55*, 712A–724A.

**(5)** Uhteg, K.; Jarrett, J.; Richards, M.; Howard, C.; Morehead, E.; Geahr, M.; Gluck, L.; Hanlon, A.; Ellis, B.; Kaur, H. Comparing the Analytical Performance of Three SARS-CoV-2 Molecular Diagnostic Assays. *J. Clin. Virol.* **2020**, *127*, 104384.

**(6)** Baek, Y. H.; Um, J.; Antigua, K. J. C.; Park, J.-H.; Kim, Y.; Oh, S.; Kim, Y.-I.; Choi, W.-S.; Kim, S. G.; Jeong, J. H. Development of a Reverse Transcription–Loop-Mediated Isothermal Amplification as a Rapid Early-Detection Method for Novel SARS-CoV-2. *Emerg. Microbes Infect.* **2020**, *9*, 998–1007.

**(7)** Zhu, X.; Wang, X.; Han, L.; Chen, T.; Wang, L.; Li, H.; Li, S.; He, L.; Fu, X.; Chen, S. Multiplex Reverse Transcription Loop-Mediated Isothermal Amplification Combined with Nanoparticle-Based Lateral Flow Biosensor for the Diagnosis of COVID-19. *Biosens. Bioelectron.* **2020**, *166*, 112437.

**(8)** Chow, F. W.-N.; Chan, T. T.-Y.; Tam, A. R.; Zhao, S.; Yao, W.; Fung, J.; Cheng, F. K.-K.; Lo, G. C.-S.; Chu, S.; Aw-Yong, K. L. A Rapid, Simple, Inexpensive, and Mobile Colorimetric Assay COVID-19-LAMP for Mass On-Site Screening of COVID-19. *Int. J. Mol. Sci.* **2020**, *21*, 5380.

**(9)** El Wahed, A. A.; Patel, P.; Maier, M.; Pietsch, C.; Rüster, D.; Böhlken-Fascher, S.; Kissenkötter, J.; Behrmann, O.; Frimpong, M.; Diagne, M. M. Suitcase Lab for Rapid Detection of SARS-CoV-2 Based on Recombinase Polymerase Amplification Assay. *Anal. Chem.* **2021**, *93*, 2627–2634.

**(10)** Behrmann, O.; Bachmann, I.; Spiegel, M.; Schramm, M.; Abd El Wahed, A.; Dobler, G.; Dame, G.; Hufert, F. T. Rapid Detection of SARS-CoV-2 by Low-Volume Real-Time Single-Tube Reverse Transcription Recombinase Polymerase Amplification Using an Exo Probe with an Internally Linked Quencher (Exo-IQ). *Clin. Chem.* **2020**, *66*, 1047–1054.

**(11)** Broughton, J. P.; Deng, X.; Yu, G.; Fasching, C. L.; Servellita, V.; Singh, J.; Miao, X.; Streithorst, J. A.; Granados, A.; Sotomayor-Gonzalez, A. CRISPR–Cas12-Based Detection of SARS-CoV-2. *Nat. Biotechnol.* **2020**, *38*, 870–874.

**(12)** Mojsoska, B.; Larsen, S.; Olsen, D. A.; Madsen, J. S.; Brandslund, I.; Alatraktchi, F. A. A. Rapid SARS-CoV-2 Detection Using Electrochemical Immunosensor. *Sensors* **2021**, *21*, 390.

**(13)** Fabiani, L.; Saroglia, M.; Galatà, G.; De Santis, R.; Fillo, S.; Luca, V.; Faggioni, G.; D'Amore, N.; Regalbuto, E.; Salvatori, P. Magnetic Beads Combined with Carbon Black-Based Screen-Printed Electrodes for COVID-19: A Reliable and Miniaturized Electrochemical Immunosensor for SARS-CoV-2 Detection in Saliva. *Biosens. Bioelectron.* **2021**, *171*, 112686.

**(14)** Chaibun, T.; Puenpa, J.; Ngamdee, T.; Boonapatcharoen, N.; Athamanolap, P.; O'Mullane, A. P.; Vongpunsawad, S.; Poovorawan, Y.; Lee, S. Y.; Lertanantawong, B. Rapid Electrochemical Detection of Coronavirus SARS-CoV-2. *Nat. Commun.* **2021**, *12*, 802.

**(15)** Zhao, H.; Liu, F.; Xie, W.; Zhou, T.-C.; Ouyang, J.; Jin, L.; Li, H.; Zhao, C.-Y.; Zhang, L.; Wei, J. Ultrasensitive Supersandwich-Type Electrochemical Sensor for SARS-CoV-2 from Infected COVID-19 Patients Using a Smartphone. *Sens. Actuators, B* **2021**, *327*, 128899.
**(16)** Rahmati, Z.; Roushani, M.; Hosseini, H.; Choobin, H. Electrochemical Immunosensor with $Cu_2O$ Nanocube Coating for Detection of SARS-CoV-2 Spike Protein. *Microchim. Acta* **2021**, *188*, 105.
**(17)** Curti, F.; Fortunati, S.; Knoll, W.; Giannetto, M.; Corradini, R.; Bertucci, A.; Careri, M. A Folding-Based Electrochemical Aptasensor for the Single-Step Detection of the SARS-CoV-2 Spike Protein. *ACS Appl. Mater. Interfaces* **2022**, *14*, 19204–19211.
**(18)** Eissa, S.; Zourob, M. Development of a Low-Cost Cotton-Tipped Electrochemical Immunosensor for the Detection of SARS-CoV-2. *Anal. Chem.* **2020**, *93*, 1826–1833.
**(19)** Shao, W.; Shurin, M. R.; Wheeler, S. E.; He, X.; Star, A. Rapid Detection of SARS-CoV-2 Antigens Using High-Purity Semiconducting Single-Walled Carbon Nanotube-Based Field-Effect Transistors. *ACS Appl. Mater. Interfaces* **2021**, *13*, 10321–10327.
**(20)** Seo, G.; Lee, G.; Kim, M. J.; Baek, S.-H.; Choi, M.; Ku, K. B.; Lee, C.-S.; Jun, S.; Park, D.; Kim, H. G. Rapid Detection of COVID-19 Causative Virus (SARS-CoV-2) in Human Nasopharyngeal Swab Specimens Using Field-Effect Transistor-Based Biosensor. *ACS Nano* **2020**, *14*, 5135–5142.
**(21)** Li, J.; Wu, D.; Yu, Y.; Li, T.; Li, K.; Xiao, M.-M.; Li, Y.; Zhang, Z.-Y.; Zhang, G.-J. Rapid and Unamplified Identification of COVID-19 with Morpholino-Modified Graphene Field-Effect Transistor Nanosensor. *Biosens. Bioelectron.* **2021**, *183*, 113206.
**(22)** Chen, H.; Park, S.-G.; Choi, N.; Kwon, H.-J.; Kang, T.; Lee, M.-K.; Choo, J. Sensitive Detection of SARS-CoV-2 Using a SERS-Based Aptasensor. *ACS Sens.* **2021**, *6*, 2378–2385.
**(23)** Shim, J.-E.; Kim, Y. J.; Choe, J.-H.; Lee, T. G.; You, E.-A. Single-Nanoparticle-Based Digital SERS Sensing Platform for the Accurate Quantitative Detection of SARS-CoV-2. *ACS Appl. Mater. Interfaces* **2022**, *14*, 38459–38470.
**(24)** Antoine, D.; Mohammadi, M.; Vitt, M.; Dickie, J. M.; Jyoti, S. S.; Tilbury, M. A.; Johnson, P. A.; Wawrousek, K. E.; Wall, J. G. Rapid, Point-of-Care scFv-SERS Assay for Femtogram-Level Detection of SARS-CoV-2. *ACS Sens.* **2022**, *7*, 866–873.
**(25)** Boumar, I.; Deliorman, M.; Sukumar, P.; Qasaimeh, M. A. Spike- and Nucleocapsid-Based Gold Colloid Assay toward the Development of an Adhesive Bandage for Rapid SARS-CoV-2 Immune Response Detection and Screening. *Microsyst. Nanoeng.* **2023**, *9*, 82.
**(26)** Wang, Z.; Zheng, Z.; Hu, H.; Zhou, Q.; Liu, W.; Li, X.; Liu, Z.; Wang, Y.; Ma, Y. A Point-of-Care Selenium Nanoparticle-Based Test for the Combined Detection of Anti-SARS-CoV-2 IgM and IgG in Human Serum and Blood. *Lab Chip* **2020**, *20*, 4255–4261.
**(27)** Derin, D. C.; Gültekin, E.; Taşkın, İ. İ.; Yakupoğulları, Y. Development of Nucleic Acid-Based Lateral Flow Assays for SARS-CoV-2 Detection. *J. Biosci. Bioeng.* **2023**, *135*, 87–92.
**(28)** Moitra, P.; Alafeef, M.; Dighe, K.; Frieman, M. B.; Pan, D. Selective Naked-Eye Detection of SARS-CoV-2 Mediated by N-Gene-Targeted Antisense Oligonucleotide-Capped Plasmonic Nanoparticles. *ACS Nano* **2020**, *14*, 7617–7627.
**(29)** Versiani, A. F.; Andrade, L. M.; Moraes, T. F.; Martins, E. M.; Bagno, F. F.; Andrade, L. A.; Campos, G. R.; dos Santos, T. M.; Moraes, M. M.; Sérgio, S. A. R. Serologic LSPR Nanosensor against SARS-CoV-2 Antibodies and Related Variants Outperforms ELISA in Sensitivity. *npj Biosensing* **2025**, *2*, 11.

**(30)** Ma, Z.; Sun, Z.; Lv, X.; Chen, H.; Geng, Y.; Geng, Z. Sensitivity-Enhanced Nanoplasmonic Biosensor Using Direct Immobilization of Two Engineered Nanobodies for SARS-CoV-2 Spike Receptor-Binding Domain Detection. *Sens. Actuators, B* **2023**, *383*, 133575.
**(31)** Qiu, G.; Gai, Z.; Tao, Y.; Schmitt, J.; Kullak-Ublick, G. A.; Wang, J. Dual-Functional Plasmonic Photothermal Biosensors for Highly Accurate Severe Acute Respiratory Syndrome Coronavirus 2 Detection. *ACS Nano* **2020**, *14*, 5268–5277.
**(32)** Qiu, G.; Gai, Z.; Saleh, L.; Tang, J.; Gui, T.; Kullak-Ublick, G. A.; Wang, J. Thermoplasmonic-Assisted Cyclic Cleavage Amplification for Self-Validating Plasmonic Detection of SARS-CoV-2. *ACS Nano* **2021**, *15*, 7536–7546.
**(33)** Qiu, G.; Spillmann, M.; Tang, J.; Zhao, Y. B.; Tao, Y.; Zhang, X.; Geschwindner, H.; Saleh, L.; Zingg, W.; Wang, J. On-Site Quantification and Infection Risk Assessment of Airborne SARS-CoV-2 Virus via a Nanoplasmonic Bioaerosol Sensing System in Healthcare Settings. *Adv. Sci.* **2022**, *9*, 2204774.
